# Global burden of asthma attributable to high body mass index in older adults 1990–2021 and prediction to 2050: An analysis of Global Burden of Disease Study 2021^☆^

**DOI:** 10.1016/j.waojou.2025.101040

**Published:** 2025-03-12

**Authors:** Zhikang Wang, Yifang Liu, Yilin Li, Qi Wang, Junan Liu

**Affiliations:** a Department of Social Medicine and Health Management, School of Public Health, Tongji Medical College, Huazhong University of Science and Technology, Wuhan, Hubei Province, China; b Department of Epidemiology and Biostatistics, School of Public Health, Tongji Medical College, Huazhong University of Science and Technology, Wuhan, 430030, China

**Keywords:** Asthma, Global Burden of Disease Study 2021, High body mass index, Older adults, Bayesian Age-Period-Cohort Prediction

## Abstract

**Background:**

Previous studies have shown that high body mass index was a primary risk factor for asthma, particularly impacting older adults. This study aimed to assess the spatial and temporal trends for asthma burden attributable to high body mass index in older adults from 1990 to 2021 and to project trends up to 2050.

**Method:**

We extracted data from the Global Burden of Disease Study 2021 for population aged over 60 years with asthma attributable to high BMI. Relevant indicators included number of deaths, disability-adjusted life years, mortality, and disability-adjusted life years rates and the rates were directly standardized. Spearman rank correlation test tested the burden against the Socio-demographic Index (SDI). Decomposition analysis was used to decompose changes in burden according to population structure, population growth, and epidemiologic changes. The Bayesian age-period-cohort model was used to predict the burden.

**Results:**

From 1990 to 2021, despite downward trends in global mortality and disability-adjusted life-year rates, global asthma deaths, and disability-adjusted life years attributable to high body mass index increase by 69% and 46%, rising to 43,628 cases (95% CI: 18,366–71 088) and 1,223,969 years (95% CI: 526,972–1 945,426). Age-standardized mortality rates and disability-adjusted life years rates were more severe in regions with lower SDI, such as Oceania. Mortality rates and disability-adjusted life-year rates increased with age, with a higher burden observed in females compared to males. Population growth had a significant impact on the increase in deaths and disability-adjusted life years from 1990 to 2021, contributing approximately 158% and 222%, respectively. Asthma deaths and disability-adjusted life years attributable to high body mass index will continue to rise to 101,252 cases and 2,941,172 years up to 2050.

**Conclusion:**

The global asthma burden due to high body mass index in older adults has risen significantly and is expected to continue this trend, highlighting the importance of developing public health strategies to address this issue.

## Introduction

Asthma is a heterogeneous disease, usually characterized by chronic airway inflammation.^1^ The incidence of asthma has risen steadily over the past 3 decades globally.^2^ It is estimated that asthma affected about 261 million people and caused 436,000 deaths in 2021.^3^^,^^4^ In addition, asthma is associated with a variety of diseases such as chronic obstructive pulmonary disease, obstructive sleep apnoea, chronic rhinosinusitis, cardiovascular disease, and more,^5^, ^6^, ^7^, ^8^ which greatly affects the patient's quality of life.

High body mass index (BMI) is commonly related to multiple adverse health outcomes, including respiratory diseases, diabetes, high blood pressure, and abnormalities of reproductive function,^9^, ^10^, ^11^, ^12^ which accounts for approximately 3.71 million deaths worldwide in 2021.^3^ High BMI was the leading risk factor for asthma, contributing to 16.94% of the asthma burden, followed by smoking and occupational asthmagens.^13^ The mechanism by which high BMI increases asthma risk may be through alterations in lung physiology and airway mechanics, sex hormone differences, modification of the immune response, and changes in the microbiome.^14^, ^15^, ^16^

More importantly, the burden of asthma attributable to high BMI particularly impacts older adults.^17^ Four studies using Global Burden of Disease Study (GBD) 2019 data reported the burden of asthma attributable to high BMI.^2^^,^^13^^,^^17^^,^^18^ Two of these studies focused more on the analysis of the asthma burden for the whole population, with a briefer analysis of the asthma burden attributable to high BMI.^2^^,^^13^ The other 2 studies did not comprehensively analyze the asthma burden attributable to high BMI in older adults.^17^^,^^18^ Therefore, the purpose of this study, based on the estimates of deaths and disability-adjusted life years (DALYs) available from GBD 2021, was to assess the spatial and temporal trends for asthma burden attributable to high BMI in older adults from 1990 to 2021, explore the drivers of changes in asthma burden and projected the future temporal trends from 2022 to 2050. We aim to provide valuable evidence for future asthma control and policy development in older adults.

## Materials and methods

### Data sources and technical route

The GBD is the largest and most detailed scientific study ever conducted to quantify health levels and trends. The data for this study were derived from GBD 2021,^19^ which calculates global, regional, and national estimates of the burden of disease indicators for 371 diseases and injuries and 88 risk factors. This study is a secondary analysis based on GBD data and is a description of the burden of asthma attributable to high BMI. We extracted data on the number of deaths, DALYs, mortality rates, and DALYs rates of asthma in older adults attributable to high BMI from 1990 to 2021. According to GBD definition, high BMI for adults (ages 20 and older) is defined as BMI greater than 20–23 kg/m^2^, and for children (age 2–19) is defined as being overweight or obese based on International Task Force standards.^20^ The older adults in this study are those who are older than 60 years of age. We selected data from 8 age groups: "60 to 64", "65 to 69", "70 to 74", "75 to 79", "80 to 84", "85 to 89", "90 to 94", and "95 plus". The technical route for this study is shown in Fig. S1.

### General methodology for estimating asthma burden

The general approach to estimating asthma burden in GBD 2021 was described in detail elsewhere.^3^^,^^4^^,^^20^ Here we provide a short overview of the process. Data from vital registration systems, surveillance systems, and verbal autopsies were input into the Cause of Death Ensemble model (a modelling tool developed by GBD) to estimate asthma mortality.^3^ The years of life lost due to premature mortality (YLLs) were calculated as the number of deaths multiplied by the standardized life expectancy at the age of death. Similarly using DisMod-MR2.1 (a Bayesian meta-regression tool), data from published literature, national surveys, disease registries, hospital records, and other sources were adjusted to estimate nonfatal data including incidence, prevalence, and years lived with disability (YLD).^4^ YLD was calculated as asthma prevalence multiplied by the severe-specific disability weights. In addition, we followed the comparative risk assessment framework to measure the attributable burde.^20^ Seven key steps were performed: estimating effect size by quantifying the relative risk; estimating exposure levels and distribution; determining the theoretical minimum level of exposure; calculating the population attributable fraction (PAF); calculating the estimates of summary exposure value; mediation factors were estimated and used to correct for PAF overestimation; calculating the attributable burden. Detailed definitions of all terms are provided in Table S1. Here we present a few important indicators. DALYs equal the sum of YLLs and YLDs. One DALY equals 1 year of healthy life lost. Socio-demographic Index (SDI) is a socio-economic indicator that reflects the state of development of a country or region, defined as the geometric mean of the 0 to 1 index of the total fertility rate up to the age of 25, the average education of the population aged 15 years and over, and the lagged distributional income per capita.^21^ Higher SDI values indicate better socio-economic conditions in that area. Based on the SDI values in GBD 2021, countries and territories are categorized into 5 categories: high SDI, high-middle SDI, middle SDI, low-middle SDI, and low SDI. The SDI reference quintiles are available in Table S2.

### Age-standardized rate and estimated annual percentage change

The purpose of age standardization is to eliminate the influence of the age structure of the population on overall rates, thus enabling comparisons of the burden of disease across time and place. The data were directly standardized by multiplying the crude rates for each age group by the population weight of that group in the standard population and then summing to obtain the age-standardized rate (ASR). The world standard population is shown in Table S3. In addition, the estimated annual percentage change (EAPC) with 95% confidence interval (CI) was introduced to further characterize the temporal trend in ASR. Assuming a linear relationship between the natural logarithm of the ASR and year: y = b_0_+b_1_x + e, where y = ln (ASR), and x = calendar year. EAPC was calculated as 100∗(eˆb_1_-1).^22^^,^^23^ If the EAPC value and lower limit are positive, the ASR shows an increasing trend. Conversely, if the EAPC value and upper limit are negative, ASR indicates a downward trend. Otherwise, it means that ASR remains stable during the period.

### Decomposition analysis and prediction analysis

We used the decomposition methods developed by Das Gupta to decompose the death number and DALYs of asthma attributable to high body mass index by population structure, population growth and epidemiologic changes.^24^ DALY is calculated as:

$${DALY}_{a,g,e,t}=\sum_{k=1}^{20}a_{k,t}*p_{t}*e_{k,t}$$

where ${DALY}_{a,g,e,t}$ represents DALYs number cumulated by population structure, population growth and epidemiologic changes in year t; $a_{k,t}$ is the proportion of population for the age group k at year t, $p_{t}$ is the population size at year t, and $e_{k,t}$ is represented by DALYs rate for a specific age group k at year t^25^.

Bayesian Age-Period-Cohort (BAPC) modelling was used to predict the number and rate of asthma deaths, and DALYs attributable to high BMI in older adults from 2022 to 2050.^26^ BAPC modelling, together with INLA framework, is used to approximate the marginal posterior distributions, which effectively avoids the mixing and convergence problems associated with the traditional Bayesian approach that relies on Markov Chain Monte Carlo sampling techniques. Details can be found in the study.^27^

### Statistical analysis

We further explored the association between age-standardized mortality rates (ASMR) and age-standardized disability-adjusted life year rates (ASDR) with SDI at the global, regional, and national levels using the Spearman rank correlation test. Additionally, we employed the locally weighted regression scatter smoothing method to model the correlation curve. In this study, estimates were derived from the mean of 1000 draws from the distribution of estimates, with 95% uncertainty intervals (UI) at the 2.5th and 97.5th percentiles of the draws. The rates were expressed per 100,000 population. Data processing, data analysis, and data visualization were all conducted using R (version 4.3.3).

## Results

### Spatiotemporal patterns of asthma DALYs attributable to high BMI in older adults from 1990 to 2021

Global DALYs for asthma in older adults attributable to high BMI grew to 1,223,969 years (95% CI: 526,972–1 945,426), accounting for 15% of global asthma DALYs, with a 46% increase from 1990 to 2021 (Table 1, Table S4). ASDR reached 113 years per 100,000 population (95% CI: 48.63–179.65) worldwide, indicating an overall downward trend, with EAPC equal to −1.50% (95% CI: 1.63%–1.36%). The parallels were in the SDI regions, ASDR showed a downward trend in high SDI, high-middle SDI, and middle SDI regions and an upward trend in low-middle SDI and low SDI regions, with the greatest increase in low-middle SDI regions and the greatest decrease in high-middle regions (Table 1).

**Table 1:** DALYs of asthma in older adults attributable to high BMI in 1990 and 2021 for Both sexes and all regions, with EAPC from 1990 to 2021.

Location	DALY number in 1990 (years)	DALY number in 2021 (years)	ASDR in 1990 (per 100 000 population)	ASDR in 2021 (per 100 000 population)	EAPC, 1990–2021 (%)
**Global**					
Both	837,417 (367,567–1335,195)	1,223,969 (526,972–1945,426)	174.91 (76.47–279.15)	113 (48.63–179.65)	−1.5 (−1.63–1.36)
Male	366,007 (162,446–587,922)	493,308 (214,850–785,432)	174.11 (76.98–280.06)	99.62 (43.34–158.58)	−1.94 (−2.08–1.8)
Female	471,409 (207,474–768,598)	730,661 (315,708–1184,799)	176.97 (77.76–288.5)	124.6 (53.83–202.04)	−1.18 (−1.3–1.05)
**SDI regions**					
High SDI	301,453 (133,824–487,309)	260,217 (114,608–428,292)	208.81 (92.67–337.52)	95.04 (41.84–156.66)	−2.53 (−2.96–2.1)
High-middle SDI	189,909 (84,480–300185)	158,264 (71,294–247,724)	152.78 (67.63–241.63)	62.39 (28.11–97.69)	−3.48 (−3.7–3.26)
Middle SDI	150,203 (62,991–237,767)	314,480 (135,330–492,869)	135.12 (56.38–214.39)	98.13 (42.18–154.16)	−1.34 (−1.49–1.19)
Low-middle SDI	140,739 (57,750–243,086)	372,779 (151,861–634,883)	209.06 (85.21–363.95)	224.46 (91.39–385.05)	0.38 (0.3–0.45)
Low SDI	53,655 (21,611–94400)	116,977 (45,635–217,791)	213.37 (85.48–380.53)	213.36 (83.12–401.2)	0.03 (−0.02–0.07)
**GBD regions**					
Andean Latin America	1647 (699–2719)	2409 (1079–4031)	70.71 (29.96–116.9)	33.69 (15.09–56.45)	−2.53 (−2.61–2.44)
Australasia	6956 (3096–11246)	7125 (3167–11470)	224.99 (100.08–363.74)	100.08 (44.44–161.22)	−2.73 (−2.99–2.46)
Caribbean	3610 (1578–5709)	6159 (2713–9885)	113.16 (49.45–178.99)	91.68 (40.39–147.16)	−1.03 (−1.21–0.85)
Central Asia	20,849 (9086–32892)	18,452 (8328–28501)	373.93 (163.02–592.44)	193.24 (87.14–299.16)	−2.83 (−3.28–2.38)
Central Europe	58,006 (25,605–92761)	29,418 (12,934–47949)	300.95 (132.62–480.83)	97.55 (42.87–159.13)	−4.04 (−4.41–3.66)
Central Latin America	16,220 (7213–25317)	14,561 (6720–22647)	176.66 (78.38–276.11)	47.62 (21.94–74.15)	−4.75 (−4.95–4.55)
Central sub-Saharan Africa	5979 (2096–14278)	16,902 (5779–44199)	242.35 (82.93–609.93)	306.26 (101.99–839.65)	0.7 (0.64–0.77)
East Asia	64,842 (27,308–108975)	103,618 (45,828–167,074)	72.27 (30.07–123.06)	39.77 (17.59–64.17)	−2.28 (−2.45–2.11)
Eastern Europe	70,094 (31,812–109300)	18,504 (8271–29188)	187.75 (85.04–292.61)	38.21 (17.07–60.25)	−6.32 (−6.68–5.95)
Eastern sub-Saharan Africa	11,365 (4670–19587)	25,879 (9859–50859)	136.85 (55.68–242.15)	142.82 (54.18–285.11)	0 (−0.05–0.04)
High-income Asia pacific	42,560 (18,476–70098)	20,534 (8772–34227)	173.39 (75.26–285.52)	30.32 (12.95–50.53)	−6.5 (−6.9–6.11)
High-income north America	79,467 (34,567–132,794)	131,395 (57,682–221,183)	171.63 (74.56–287.1)	148.68 (65.19–250.45)	0.55 (−0.09–1.2)
North Africa and middle east	78,903 (33,220–126,694)	134,607 (61,679–205216)	430.91 (179.88–697.57)	277.08 (126.06–424.34)	−1.67 (−1.77–1.57)
Oceania	2384 (978–4319)	5059 (2098–9482)	828.77 (343.67–1503.11)	683.3 (282.78–1274.34)	−0.78 (−0.86–0.7)
South Asia	108,370 (42,216–203305)	381,782 (152,227–691,681)	172.81 (66.58–326.87)	221.62 (88.13–405.15)	1.07 (0.93–1.21)
Southeast Asia	45,719 (19,237–76068)	112,991 (47,889–183,472)	162.86 (68.48–271.16)	148.94 (63.38–242)	−0.42 (−0.56–0.29)
Southern Latin America	11,846 (5253–19251)	16,892 (7522–27691)	199.5 (88.3–324.45)	149.81 (66.69–245.64)	−1.37 (−1.55–1.2)
Southern sub-Saharan Africa	10,912 (4438–19300)	26,251 (11,899–41632)	360.72 (146.6–646.46)	404.98 (182.8–644.58)	0.19 (−0.36–0.76)
Tropical Latin America	9721 (4309–15668)	16,566 (7336–26305)	91.89 (40.67–148.42)	51.88 (22.92–82.4)	−2.49 (−2.8–2.19)
Western Europe	164,911 (73,125–268,311)	90,338 (39,318–148,367)	217.61 (96.45–354.4)	75.72 (32.91–124.95)	−3.68 (−3.96–3.39)
Western sub-Saharan Africa	23,056 (9726–37803)	44,529 (18,854–72458)	233.86 (98.74–383.36)	215.53 (91.48–352.32)	−0.26 (−0.34–0.18)

Abbreviations: DALYs, disability-adjusted life years; ASDR, age-standardized DALYs (disability-adjusted life years) rate; EAPC, estimated annual percentage change.

**Fig. 1:**
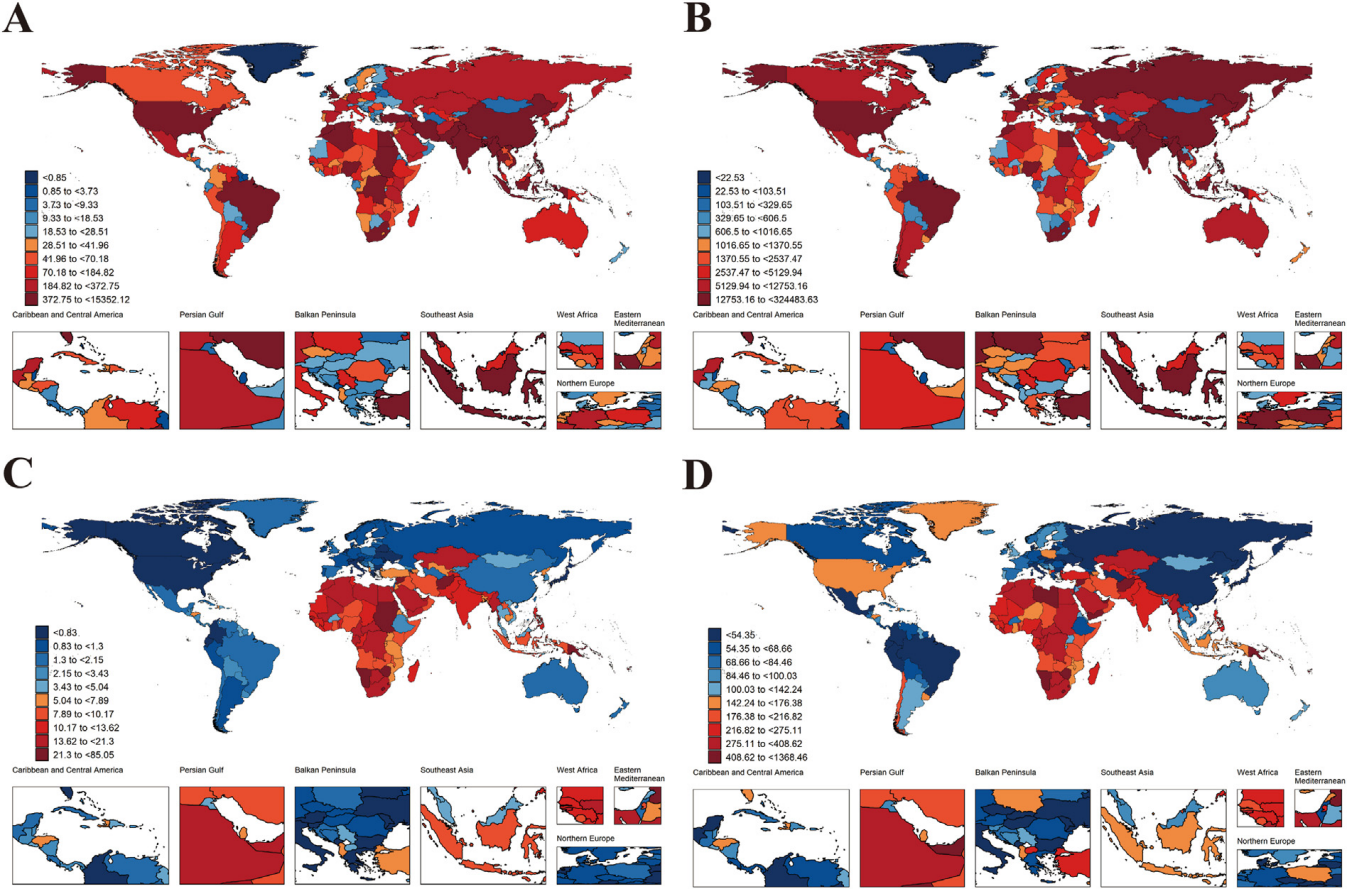
The global distribution of asthma burden in older adults attributable to high BMI for both sexes in 2021. Deaths number (A), DALYs (B), ASMR (C), and ASDR (D) of asthma in older adults attributable to high BMI for both sexes in 204 countries and territories in 2021. Abbreviations: ASMR, age-standardized mortality rate; ASDR, age-standardized DALYs (disability-adjusted life years) rate

Within the 21 GBD regions, the highest ASDR was in Oceania, at 683.30 years per 100,000 population (95% CI: 282.78–1274.34). In contrast, the lowest was in High-income Asia Pacific, at 30.32 years per 100,000 population (95% CI: 12.95–50.53). South Asia and Central Sub-Saharan Africa were showing upward trends, with South Asia showing the fastest increase (Table 1). Among countries, the difference in ASDR was close to 72-fold. Fiji's ASDR was almost 500 years per 100,000 population higher than second-placed Kiribati, at 1368.46 years per 100,000 population (95% CI: 592.12–2335.34). Ukraine had the lowest ASDR at 19.15 years per 100,000 population (95% CI: 8.26–31.88), followed by Armenia and Ecuador. Trends in ASDR also varied considerably over countries, with the largest decrease in the Republic of Korea and the largest increase in Zimbabwe (Table S6, Fig. 1D).

### Spatiotemporal patterns of asthma deaths attributable to high BMI in older adults from 1990 to 2021

From 1990 to 2021, the number of deaths of asthma in older adults attributable to high BMI increased by 69% globally to 43,628 cases (95% CI: 18,366–71 088), accounting for 14% of global asthma deaths (Table S4). ASMR decreased to 4.14 cases per 100,000 population (95% CI: 1.74–6.74), with EAPC equal to −1.26% (95% CI: 1.36–1.17) (Table S5). Of the 5 SDI regions, the Low-middle SDI region had the highest number of deaths at 17,093 cases (95% CI: 6904–30 212), while the High SDI region had the lowest at 3435 cases (95% CI: 1587–5455) in 2021. In addition, the ASMR in High SDI, High-middle SDI, and Middle SDI regions all indicated a decreasing trend, whereas Low-middle SDI and Low SDI regions indicated an increasing trend (Table S5).

Within the 21 GBD regions, the highest ASMR was in Oceania, at 36.91cases per 100,000 population (95% CI: 15.11–69.74), and the lowest was in High-income Asia Pacific, at 0.66 cases per 100,000 population (95% CI: 0.28–1.13) (Table S5). The enormous cross-country variance in ASMR has exceeded 386-fold, with Fiji having a high ASMR of more than 80 cases per 100,000 population, whereas Monaco has only 0.22 cases per 100,000 population (95% CI: 0.09–0.42). Meanwhile, ASMR decreased most dramatically in Korea and increased considerably in Belarus. What is visible is that India's death toll reached 3723 cases (95% CI, 1362 to 7393), well ahead of second-placed China (Fig. 1C–Table S7).

### Gender and age patterns of asthma burden attributable to high BMI in older adults in 2021

**Fig. 2:**
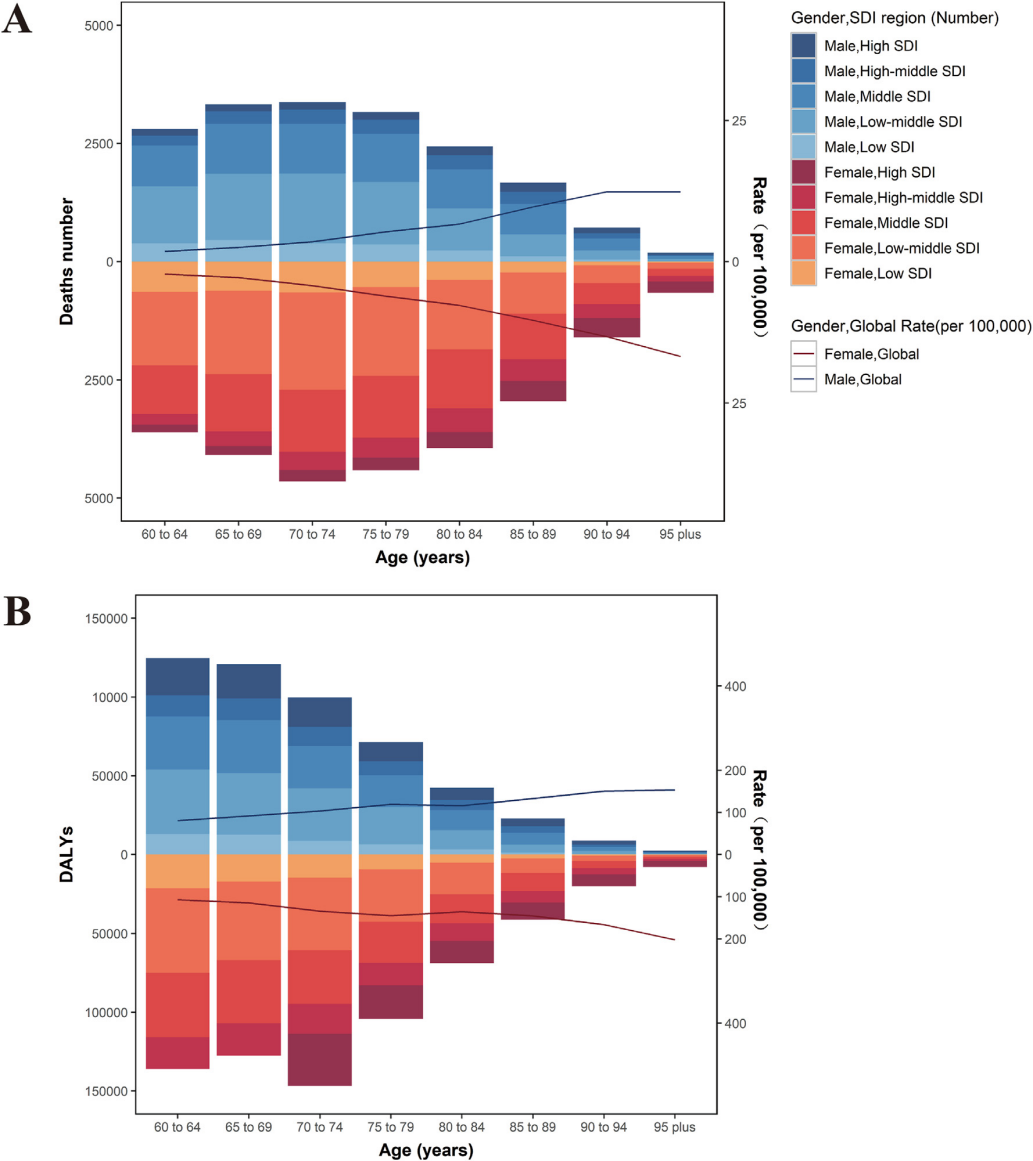
Number and age-specific rates of deaths and DALYs of asthma attributable to high BMI in older adults, by sex, in 2021. Deaths number in 5 SDI regions and global mortality (A) of asthma attributable to high BMI in older adults in different age groups, by sex, in 2021. DALYs in 5 SDI regions and global DALY rates (B) of asthma attributable to high BMI in older adults in different age groups, by sex, in 2021. Abbreviations: DALYs, disability-adjusted life years

Fig. 2 shows the number of asthma deaths and DALYs attributable to high BMI in 5 SDI regions for males and females in 8 age groups in 2021 and the age-specific rates of global mortality and DALYs rates. The number of age-specific deaths for both males and females showed an unimodal distribution, with a peak in the″70 to 74″ age group and a major concentration in the middle SDI and low-middle SDI regions. The global mortality rates showed an increasing trend with age. Unlike the deaths, the age-specific DALYs declined with age, but the highest values in the ″70 to 74″ age group occurred in females. It is noteworthy that DALYs remained higher in lower SDI regions (Fig. 2B). The global age-specific DALY rates for both males and females increased slowly with age, reaching a maximum in the ″95 plus″ age group.

### Association of asthma burden attributable to high BMI in older adults with socio-demographic index

**Fig. 3:**
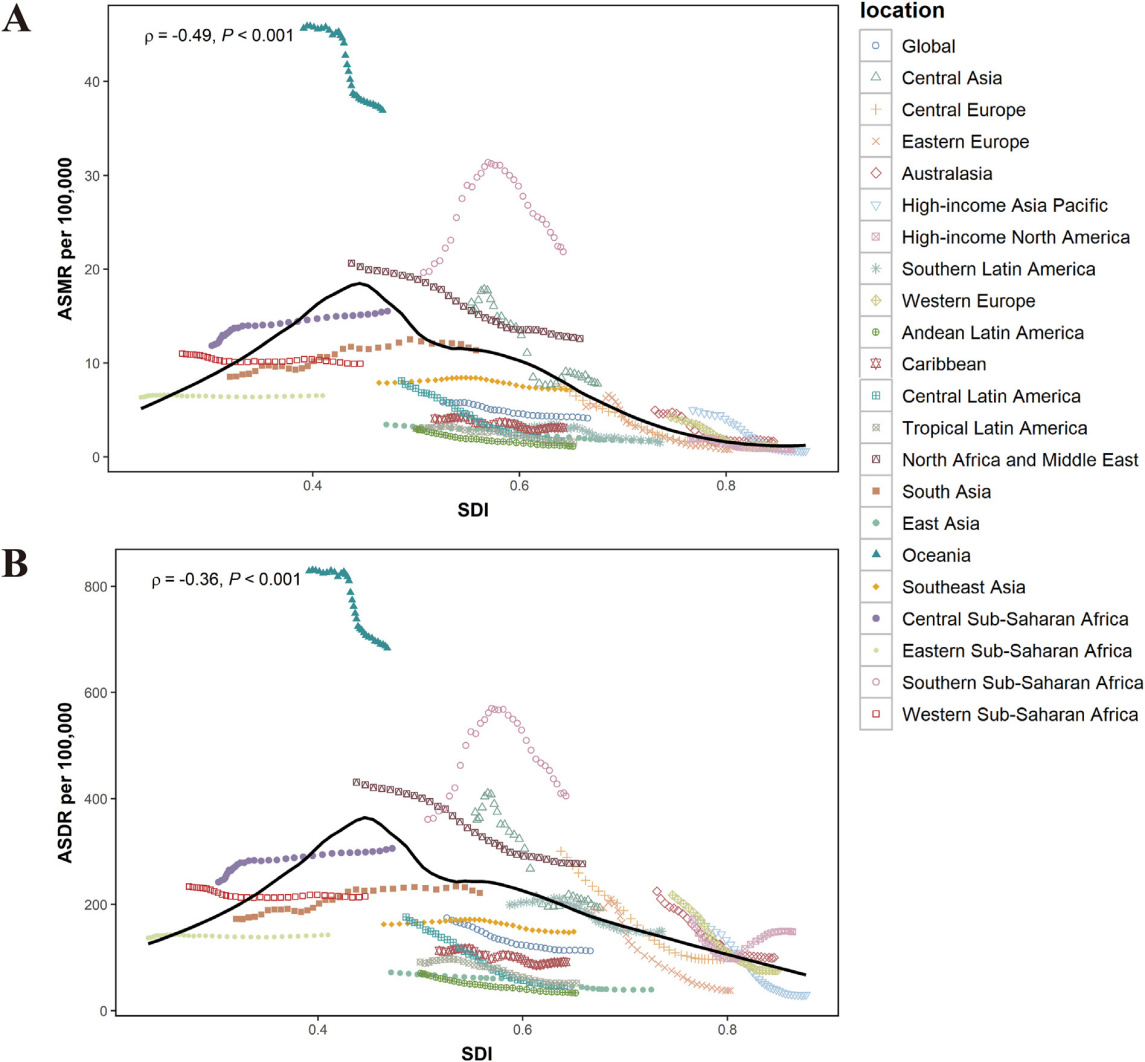
Association of ASR of asthma in older adults attributable to high BMI with SDI at the regional level. ASMR (A) and ASDR (B) of asthma in older adults attributable to high BMI at the global level and in 21 GBD regions, by SDI, from 1990 to 2021. The black line in the figure represents the expectation ASR based on SDI. Abbreviations: ASMR, age-standardized mortality rate; ASDR, age-standardized DALYs (disability-adjusted life years) rate; ASR, age-standardized rate; SDI, Socio-demographic Index

Fig. 3 shows the observed versus expected ASMR and ASDR at the regional level from 1990 to 2021 based on SDI value. ASR and SDI were negatively correlated, indicating that the asthma burden decreased with growing SDI values. As the SDI values changed, the trends of ASMR and ASDR were essentially consistent. Most of the regions with lower SDI levels showed a slowly increasing trend in asthma burden in older adults attributed to high BMI, while most of the regions with higher SDI indicated a decreasing trend (Fig. 3). The observed ASMR and ASDR at the global level were both lower than expected. However, it is worth noting that the observed ASMR and ASDR in Oceania and Southern Sub-Saharan Africa were both much higher than expected. Fig. S2 shows the observed versus expected ASMR and ASDR at the national level in 2021 based on SDI value. It is visible that the observed ASMR and ASDR in Fiji were higher than the expected level.

### Decomposition analysis of the change in asthma burden attributable to high BMI in older adults from 1990 to 2021

**Fig. 4:**
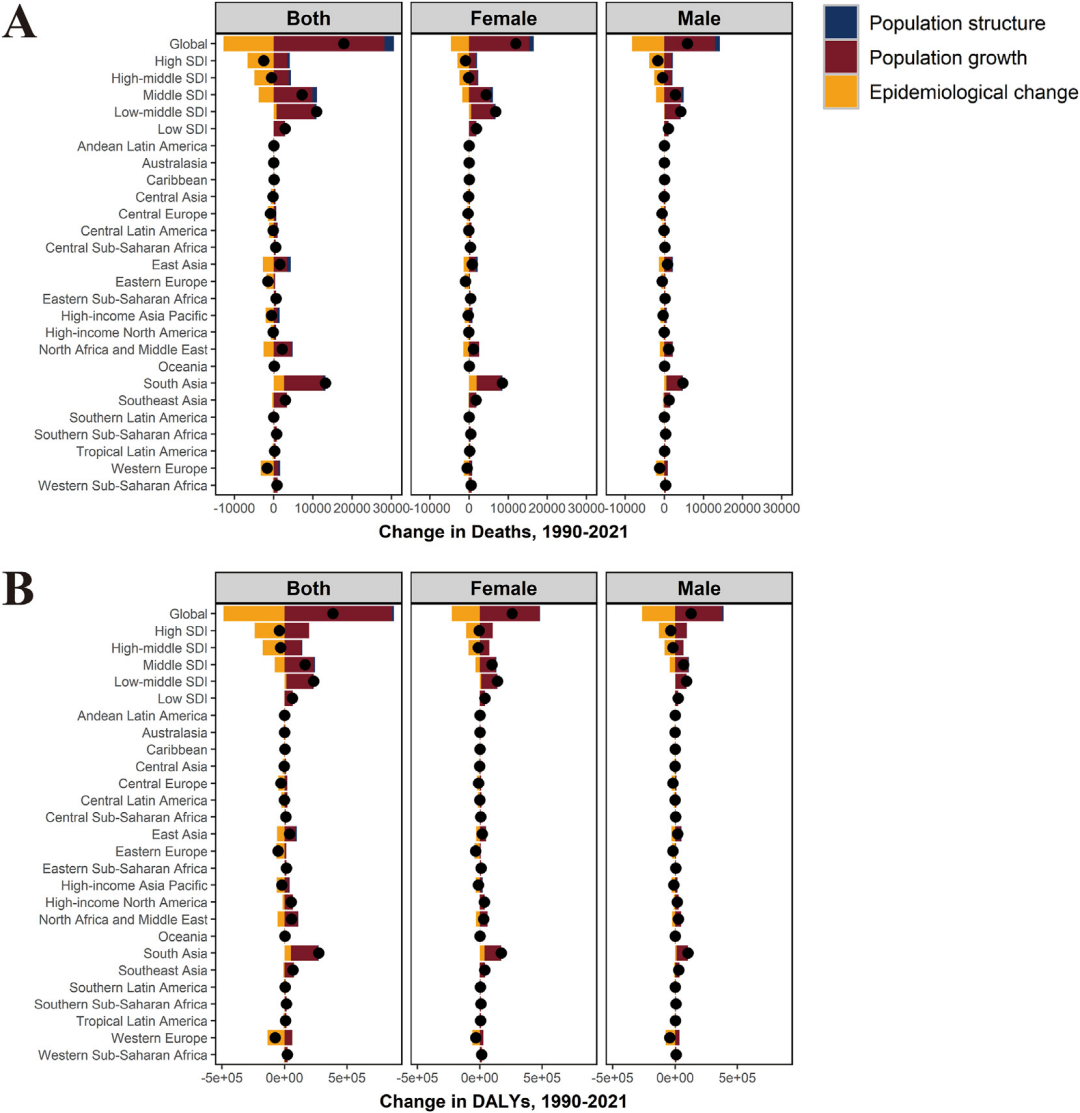
Decomposition analysis of the change in number of deaths and DALYs. Change in number of deaths (A) or DALYs (B) of asthma in older adults attributable to high BMI decomposed by 3 population-level drivers: population structure, population growth, and epidemiologic change from 1990 to 2021 at the global level and in 5 SDI regions and 21 GBD regions. The black dot in the figure represents the total change of DALYs or number of deaths or number of deaths. Abbreviations: DALYs, disability-adjusted life years

Fig. 4 shows the results of the decomposition analysis regarding the contribution of 3 population-level drivers: population aging, population growth, and epidemiological changes to the change in DALYs and number of death in older adults from 1990 to 2021, at the global level and in SDI regions and GBD regions. Population growth contributed 222.37% to the global increase in DALYs for asthma in older adults attributable to high BMI, followed by demographics (3.76%), while epidemiologic changes (−126.13%) contributing to the reduction in DALYs (Table S8). Changes in DALYs for males and females were not significantly different in the distribution patterns of the 3 drivers and were similar to the full global population (Fig. 4B). At the SDI regional level, population growth played an important role in the growth of DALYs in all SDI regions. Epidemiologic changes (changes in ASDR) contributed to the reduction of DALYs in high SDI, high-middle SDI, and middle SDI regions, especially in high SDI regions. However, the impact of epidemiologic changes causing a decrease in DALYs was offset by population growth effects in middle SDI, showing an overall net increase in DALYs (Fig. 4B–Table S8). In addition, middle SDI, low-middle SDI, and low SDI regions indicated positive growth. Among the GBD regions, the highest increase in asthma burden among the elderly attributable to high BMI was observed in South Asia, with all 3 population-level drivers showing positive effects. In the high SDI regions, negative growth due to epidemiologic changes offset the positive effects of population aging and population growth, achieving a relatively pronounced decline in the number of deaths (Fig. 4A–Table S9).

### Prediction of asthma burden attributable to high BMI in older adults from 2022 to 2050

**Fig. 5:**
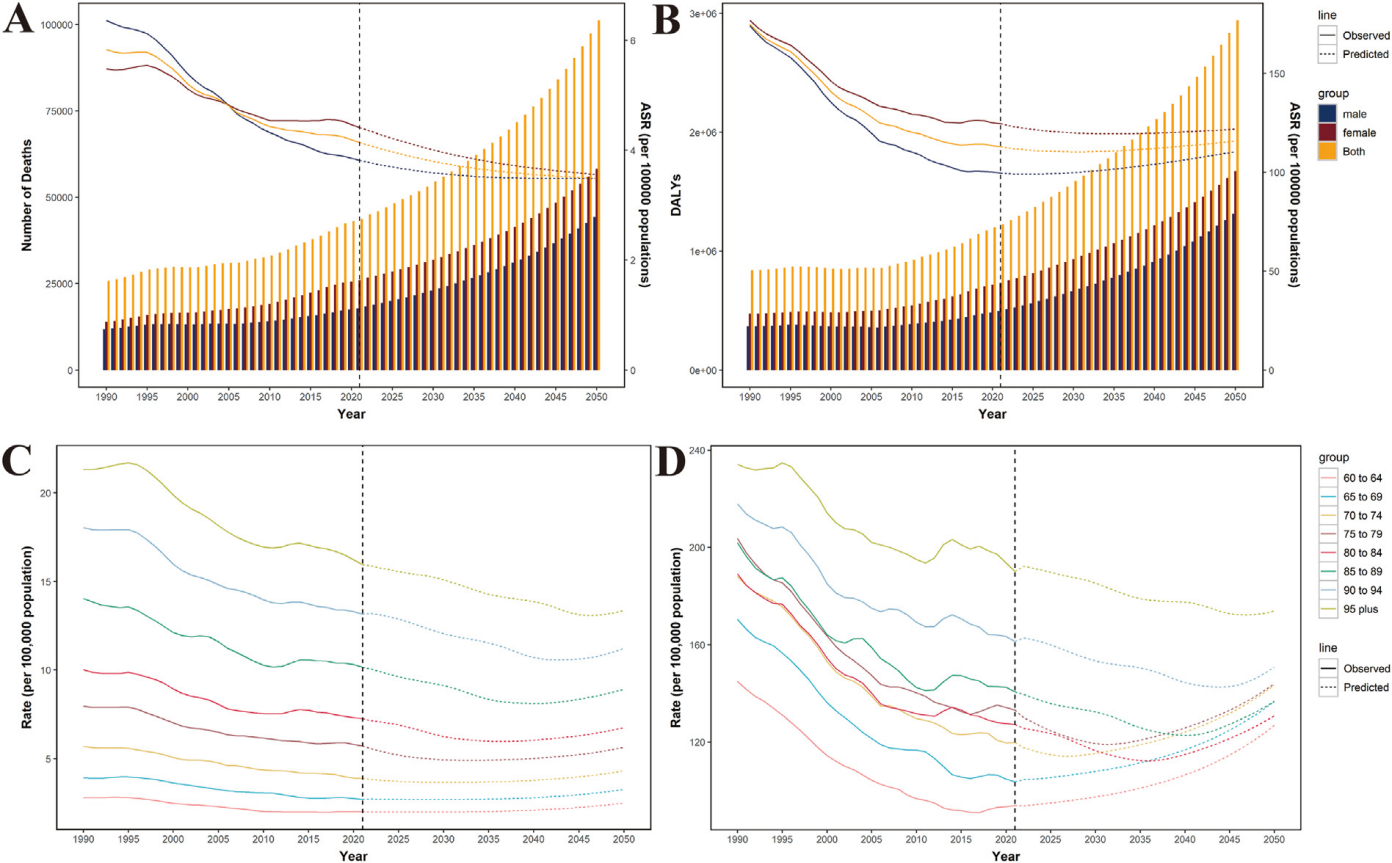
Prediction of asthma in death number, ASMR and DALYs, ASDR among older adults. Prediction of death number and ASMR (A), DALYs, and ASDR (B). Prediction of the mortality (C) and DALY rate (D) for 8 different age groups. Column length represents number and line represents ASR. Abbreviations: DALYs, disability-adjusted life years; ASDR, age-standardized DALYs (disability-adjusted life years) rate; ASR, age-standardized rate

Fig. 5 showed the prediction of the number and ASR of deaths and DALYs, and also showed the prediction of the mortality and DALY rate for 8 different age groups using the BAPC model. Trends in observed and predicted asthma burden in older adults attributable to high BMI varied between genders, with females higher than males. Asthma deaths and disability-adjusted life years attributable to high BMI will continue to rise to 101,252 cases and 2,941,172 years up to 2050 (Table S10, Table S11). ASMR will show a downward trend, but ASDR will remain stable (Fig. 5B–Table S12, Table S13). The mortality rates for the 8 age groups will maintain a downward trend followed by a slow rise. The disability-adjusted life-year rates will maintain a rapid upward trend in “60–64”, “65 to 69″, and “70 to 74″ age groups, and the other groups will maintain a downward trend followed by a rise (Fig. 5D–Table S14, Table S15).

## Discussion

This study comprehensively assessed the asthma burden attributable to high BMI in older adults by analyzing GBD2021 data on deaths, and DALYs from 1990 to 2021. Despite a decline in asthma ASMR/ASDR in older adults attributed to high BMI, the number of deaths and DALYs increased substantially. In addition, the number and rate of asthma deaths and DALYs vary by gender, country, and age group. The results of the decomposition analysis indicated that the rapid growth of older adults was the most important factor contributing to the increased burden of asthma attributable to high BMI in older adults.

Previous GBD studies had focused more on the global and regional burden of asthma or high BMI.^2^^,^^13^^,^^28^ However, there were fewer studies on the burden of asthma attributable to high BMI,^17^ especially in high-burden older adults. Previous studies have some similar findings to this study. For example, an increase in the number of asthma deaths over 3 decades despite decreases in age-standardized asthma mortality rates.^2^^,^^13^ The burden of asthma was more severe in the population over 60 years of age, in children under 9 years of age, and females.^2^^,^^13^^,^^17^ There was a negative correlation between ASDR of asthma and SDI.^2^^,^^13^ However, the study by Zhang et al reported higher asthma DALYs in children attributable to high BMI in males than in females in China and the United States,^18^ which was contrary to the results of this study in older adults. This may be linked to sex hormone levels in adult females,^29^^,^^30^ which may affect immune system function, making females more susceptible to asthma.

Global Strategy for Asthma Management and Prevention states that being overweight or obese affects asthma risk in adults, while obesity can lead to poor asthma control.^1^ According to the definition by the European Respiratory Society (ERS) and the American Thoracic Society (ATS), severe asthma in individuals over 6 years of age is defined as “asthma which requires treatment with guidelines suggested medications for GINA steps 4–5 asthma (high-dose inhaled corticosteroids (ICS) and long-acting beta2 agonist (LABA) or leukotriene modifier/theophylline) for the previous year or systemic CS for ≥50% of the previous year to prevent it from becoming uncontrolled or which remains uncontrolled despite this therapy".^31^ Severe asthma includes refractory asthma, which is poorly controlled disease despite optimal therapy.^32^ And severe asthma is often associated with multimorbidity,^1^ including obesity, which can further complicate asthma management. Multiple studies have also showed that overweight or obesity causes asthma to occur and develop.^33^, ^34^, ^35^, ^36^, ^37^ However, the mechanism is currently not elucidated and may be realized through the following pathways: airway and systemic oxidative stress;^33^ microbial flora imbalance;^38^ enhancement of the inflammatory response;^39^ altered immune cell phenotype and corticosteroid pharmacokinetics.^40^ In addition, the increasing ageing of the world's population and the deterioration of the respiratory and immune systems may be important reasons for the high burden on older adults.^41^^,^^42^

Asthma burden attributable to high BMI in older adults was more severe in lower SDI areas represented by Oceania and Southern Sub-Saharan Africa. On the contrary, the higher SDI regions represented by High-income Asia Pacific and Western Europe had a lower burden and showed a decreasing trend. A study also showed that there are geographic differences in asthma incidence, with prevalence increasing with decreasing socioeconomic status.^43^ The results of the decomposition analyses indicated that the growth of the elderly population was the main reason for the increase in burden in the lower SDI areas, accompanied by poor asthma control. The GBD 2016 Healthcare Access and Quality Collaborators published a study in the Lancet to quantify the level of healthcare across countries and regions, in which the Healthcare Access and Quality (HAQ) Index was constructed to assess individual healthcare accessibility and quality, with lower SDI regions also having lower HAQ index.^44^ In developed regions, where medical resources are abundant, the prevention, diagnosis, and prognosis of asthma tend to be specialized and timely, and more attention is paid to the health care of the elderly. However, a Lancet study suggested that the burden of obesity was worse in these regions.^45^ This partly suggests that the development of better health policies can be effective in relieving the burden of asthma in the elderly attributable to high BMI. At the country level, we can observe that India has the heaviest absolute asthma burden attributable to high BMI in older adults. Apart from the above reason, India has a large elderly population base, and Indian asthma patients have bad healthcare behavior and poor treatment compliance due to social, economic, religious cultural, and educational factors.^46^^,^^47^

At the same time, we noticed that the burden of asthma in older adults attributable to high BMI was higher in females than in males. This gender difference can be explained by discrepancies in physiological and hazard exposure levels. First of all, the prevalence of obesity is higher in females than in males,^48^ and under the same BMI conditions, women's total body fat content is significantly higher than that of men.^49^ Kaisinger et al. noted that the effect of particular genes on the risk of obesity depends on sex and age, and the mutations in 3 genes, DIDO1, PTPRG, and SLC12A5, were associated with higher BMI in adult women.^50^ In addition, obesity is associated with poorer asthma control in females.^50^ The higher prevalence of asthma comorbidities in women also may result in increased asthma symptoms and a reduction in the efficacy of asthma treatment.^51^

Our projections showed that the absolute asthma burden attributable to high BMI in older adults would maintain a continuing upward trend from 2022 to 2050, which would result in a significant economic and health burden. This growing trend not only poses a threat to the quality of life of older people, but also has the potential to exacerbate the strain on healthcare resources and lead to greater pressure on public health systems. Therefore, there is an urgent need for effective prevention and intervention measures. For example, promoting healthy diets and increased physical activity,^52^^,^^53^ Global Strategy for Asthma Management and Prevention also recommends that ICS-containing treatment should be initiated when (or as soon as possible after) the diagnosis of asthma is made.^1^ At the same time, policymakers may consider promoting healthy lifestyles among residents through financial incentives.^54^ The community can also organize regular health promotion activities to engage and encourage older patients to participate in obesity control and asthma self-management.^55^

The strengths of this study are the following, firstly, the GBD data integrates a large amount of health data globally, and the accuracy and reliability of the data are high, so the study has high scientific value. Secondly, we conducted the first comprehensive assessment of asthma burden in older adults attributable to high BMI, which provided evidence for asthma control and policy development in older adults; thirdly, compared with other GBD studies on the same topic, we conducted predictive analyses of the burden, and the results showed an upward trend, suggesting the necessity of strengthening asthma and obesity control in older adults.

However, it has to be recognized that there are some limitations to this study. Firstly, although the GBD study took a variety of approaches to calculating the estimates, the data itself is potentially uncertain. Secondly, the accuracy and completeness of data collected in different countries may vary considerably, thus affecting GBD estimates.

## Conclusion

Between 2019 and 2021, despite a downward trend in ASMR and ASDR for older adults attributable to high BMI, the number of deaths and DALYs continued to rise rapidly. Growth in the older population was a determinant of the change in absolute burden of asthma attributable to high BMI. In 2021, there were regional and gender differences in the burden of asthma in older adults attributable to high BMI, with lower SD regions, represented by Oceania and Southern Sub-Saharan Africa, typically having higher burdens. Meanwhile India had the highest absolute burden of asthma attributable to high BMI, far exceeding other countries. Global burden of asthma attributable to high BMI is higher for older females than for males. This suggests that we should take into account gender differences and regional characteristics to adopt personalized lifestyle interventions and medical treatments. Simultaneously the projections showed that the burden of asthma in older adults attributable to high BMI will continue to remain upward, suggesting that it is even more urgent to control body weight in older adults and to strengthen the management of asthma interventions in older patients.

## Abbreviations

ASR, age-standardized rate; ASMR, age-standardized mortality rates; ASDR, age-standardized DALYs (disability-adjusted life years) rates; BMI, body mass index; DALYs, disability -adjusted life years; EAPC, the estimated annual percentage change; GBD, Global Burden of Disease; SDI, Socio-demographic Index; YLLs, the years of life lost due to premature mortality; YLDs, the years lived with a disability.

## Data sharing statement

The data used in this study can be obtained online (https://ghdx.healthdata.org/).

## Author contributions

Zhikang Wang: Methodology, Formal analysis, Visualization and Writing- Original draft; Yifang Liu: Methodology, Data curation, and Writing- Reviewing & Editing; Yilin Li: Formal analysis, Data curation, and Writing- Reviewing & Editing; Qi Wang: Methodology and Writing- Reviewing; Junan Liu: Conceptualization, Supervision, Funding Acquisition, and Writing- Reviewing & Editing.

## Ethics statement

We confirm that this study has not been submitted elsewhere and is original. No AI tools were used for data analysis or image generation; all work was conducted manually. The research is accurately described, and sufficient detail is provided for reproducibility. We uphold honesty and avoid any fraudulent statements.

## Publication consent statement

All authors declare that this manuscript is an original work and has not been published or submitted elsewhere. We consent to submit this manuscript to “*World Allergy Organization Journal*” for consideration for publication and grant the journal the right to publish it upon acceptance.

## Funding

This work was funded by the 10.13039/501100001809National Natural Science Foundation of China [Grant no. 72274070].

## Declaration of competing interest

The authors report no competing interests.
