# Supplementary material for: Global burden of asthma attributable to high body mass index in older adults 1990–2021 and prediction to 2050: An analysis of Global Burden of Disease Study 2021
Source: World Allergy Organ J. 2025 Mar 12;18(3):101040. doi: 10.1016/j.waojou.2025.101040 (PMC11946875; doi:10.1016/j.waojou.2025.101040)
Supplement: Multimedia component 1 [file mmc1.docx]

**Global Burden of Asthma Attributable to High Body Mass Index in Older Adults 1990-2021 and Prediction to 2050: An Analysis of Global Burden of Disease Study 2021**

**Supplementary Information**

[**Table S1.** Term definitions 3](#_Toc188728635)

[**Table S2.** SDI reference quintiles 5](#_Toc188728636)

[**Table S3.** GBD world population age standard 6](#_Toc188728637)

[**Table S4.** Percent of asthma burden attributable to high BMI at global and regional levels 7](#_Toc188728638)

[**Table S5.** Deaths of asthma in older adults attributable to high BMI in 1990 and 2021 for both sexes and all regions, with EAPC from 1990 to 2021. 9](#_Toc188728639)

[**Table S6.** DALYs of asthma in older adults attributable to high BMI in 1990 and 2021 for all nations, with EAPC from 1990 to 2021. 13](#_Toc188728640)

[**Table S7.** Deaths of asthma in older adults attributable to high BMI in 1990 and 2021 for all nations, with EAPC from 1990 to 2021. 34](#_Toc188728641)

[**Table S8.** Decomposition analysis of the change in DALYs 55](#_Toc188728642)

[**Table S9.** Decomposition analysis of the change in death number 57](#_Toc188728643)

[**Table S10.** Global asthma disability-adjusted life years attributable to high body mass index in older adults predicted to 2050 by the BAPC model across genders 59](#_Toc188728644)

[**Table S11.** Global number of asthma deaths attributable to high body mass index in older adults predicted to 2050 by the BAPC model across genders 60](#_Toc188728645)

[**Table S12.** Global asthma age-standardized mortality rates attributable to high body mass index in older adults predicted to 2050 by the BAPC model across genders 61](#_Toc188728646)

[**Table S13.** Global asthma age-standardized disability-adjusted life year rates attributable to high body mass index in older adults predicted to 2050 by the BAPC model across genders 62](#_Toc188728647)

[**Table S14.** Global asthma mortality rate attributable to high body mass index in older adults in each age group predicted by the BAPC model to 2050 63](#_Toc188728648)

[**Table S15.** Global asthma disability-adjusted life years rate attributable to high body mass index in older adults in each age group predicted by the BAPC model to 2050 65](#_Toc188728649)

[**Fig. S1.** Technical roadmap 67](#_Toc188728650)

[**Fig. S2.** Association of ASR of asthma in older adults attributable to high BMI with SDI at national level. 68](#_Toc188728651)

**Table S1.** Term definitions

| **term** | **definition** |
| --- | --- |
| Age group | A population segment within a specified age range. |
| Deaths | Deaths occurring in a population during a certain time period. |
|  |  |
| Disability-adjusted life years (DALYs) | DALY is an abbreviation for disability-adjusted life year. It is a universal metric that allows researchers and policymakers to compare very different populations and health conditions across time. DALYs equal the sum of years of life lost (YLLs) and years lived with disability (YLDs). One DALY equals one lost year of healthy life. DALYs allow us to estimate the total number of years lost due to specific causes and risk factors at the country, regional, and global levels. |
| Location | Includes country, non-sovereign region, principal administrative unit of a country (e.g., state, province), GBD region, or other custom administrative division. |
| Risk (risk factor) | An attribute, behavior, exposure, or other factor which is causally associated with an increased (or decreased) probability of a disease or injury. If the probability decreased, the risk is a protective factor. |
| Risk-attributable deaths or DALYs | The share of deaths or DALYs that can be attributed to – i.e., estimated to occur due to – exposure to a particular risk factor (e.g., alcohol-attributable deaths, or deaths attributable to air pollution, etc.) |
| Sex | Male, female, or both sexes combined. |
| Socio-demographic Index (SDI) | SDI is an abbreviation for Socio-demographic Index, a summary measure that identifies where countries or other geographic areas sit on the spectrum of development. SDI was constructed based on three measures: i) lag-distributed income per capita; ii) average years of schooling in ages 15 and older; and iii) total fertility rate (TFR) for females under age 25.For example, a low SDI value will be assigned to a country with lower income per capita, fewer average years of schooling, and higher TFR relative to other countries; conversely, a higher SDI value will be assigned to a country with higher income per capita, greater average years of schooling, and lower TFR relative to other countries. |
| Uncertainty intervals | A range of values that reflects the certainty of an estimate. In GBD, every estimate is calculated 1,000 times, each time sampling from distributions rather than point estimates for data inputs, data transformations and model choice. The 95th uncertainty interval is determined by the 25th and 975th value of the 1,000 values after ordering them from smallest to largest. Larger uncertainty intervals can result from limited data availability, small studies, and conflicting data, while smaller uncertainty intervals can result from extensive data availability, large studies, and data that are consistent across sources. |
| Year | The period of 365 days (or 366 days in leap years) in the Gregorian calendar divided into 12 months beginning with January and ending with December. |
| Years lived with disability (YLDs) | YLD is an abbreviation for years lived with disability, which can also be described as years lived in less than ideal health. This includes conditions such as influenza, which may last for only a few days, or epilepsy, which can last a lifetime. It is measured by taking the prevalence of the condition multiplied by the disability weight for that condition. Disability weights reflect the severity of different conditions and are developed through surveys of the general public. |
| Years of life lost (YLLs) | Years of life lost (YLLs) are years lost due to premature mortality. Invented by researcher Mary Dempsey, YLLs are calculated by subtracting the age at death from the longest possible life expectancy for a person at that age. For example, if the longest life expectancy for men in a given country is 75, but a man dies of cancer at 65, this would be 10 years of life lost due to cancer. |

**Table S2.** SDI reference quintiles

| **location** | **Lower bound** | **Upper bound** |
| --- | --- | --- |
| Low SDI | 0 | 0.46581580319161997 |
| Low-middle SDI | 0.46581580319161997 | 0.6188294452454329 |
| Middle SDI | 0.6188294452454329 | 0.7119746219361235 |
| High-middle SDI | 0.7119746219361235 | 0.8102959891918925 |
| High SDI | 0.8102959891918925 | 1 |

**Table S3.** GBD world population age standard

| **Age Group** | **Percent of Population** | **Rounded** |
| --- | --- | --- |
| Early Neonatal | 0.0396232 | 0.04 |
| Late Neonatal | 0.117777 | 0.12 |
| 1 to 5 months | 0.864776 | 0.86 |
| 6 to 11 months | 1.01009 | 1.01 |
| 12 to 23 months | 2.01613 | 2.02 |
| 2 to 4 | 5.9934 | 5.99 |
| 5 to 9 | 9.65824 | 9.66 |
| 10 to 14 | 8.99361 | 8.99 |
| 15 to 19 | 8.28913 | 8.29 |
| 20 to 24 | 7.80122 | 7.8 |
| 25 to 29 | 7.59144 | 7.59 |
| 30 to 34 | 7.32171 | 7.32 |
| 35 to 39 | 6.82805 | 6.83 |
| 40 to 44 | 6.14735 | 6.15 |
| 45 to 49 | 5.51133 | 5.51 |
| 50 to 54 | 4.91312 | 4.91 |
| 55 to 59 | 4.34586 | 4.35 |
| 60 to 64 | 3.68223 | 3.68 |
| 65 to 69 | 2.98509 | 2.99 |
| 70 to 74 | 2.26526 | 2.27 |
| 75 to 79 | 1.59758 | 1.6 |
| 80 to 84 | 1.09729 | 1.1 |
| 85 to 89 | 0.604519 | 0.6 |
| 90 to 94 | 0.246663 | 0.25 |
| 95 plus | 0.0785092 | 0.08 |

**Table S4.** Percent of asthma burden attributable to high BMI at global and regional levels

| **Location** | **Percent (Deaths)** | **Percent (DALYs)** |
| --- | --- | --- |
| Global | 0.14 (0.06-0.22) | 0.15 (0.08-0.23) |
| High SDI | 0.26 (0.12-0.39) | 0.25 (0.13-0.36) |
| High-middle SDI | 0.23 (0.1-0.35) | 0.21 (0.11-0.31) |
| Middle SDI | 0.17 (0.07-0.26) | 0.16 (0.08-0.23) |
| Low-middle SDI | 0.12 (0.05-0.19) | 0.12 (0.06-0.19) |
| Low SDI | 0.11 (0.05-0.18) | 0.1 (0.05-0.15) |
| High-income Asia Pacific | 0.14 (0.06-0.22) | 0.14 (0.07-0.2) |
| High-income North America | 0.32 (0.15-0.48) | 0.28 (0.15-0.39) |
| Western Europe | 0.27 (0.12-0.41) | 0.23 (0.11-0.35) |
| Australasia | 0.3 (0.14-0.45) | 0.25 (0.13-0.36) |
| Andean Latin America | 0.24 (0.11-0.37) | 0.16 (0.1-0.23) |
| Tropical Latin America | 0.27 (0.12-0.41) | 0.19 (0.11-0.26) |
| Central Latin America | 0.29 (0.13-0.43) | 0.2 (0.11-0.28) |
| Southern Latin America | 0.31 (0.14-0.46) | 0.26 (0.14-0.37) |
| Caribbean | 0.19 (0.09-0.29) | 0.15 (0.08-0.21) |
| Central Europe | 0.3 (0.14-0.45) | 0.23 (0.12-0.34) |
| Eastern Europe | 0.31 (0.14-0.47) | 0.23 (0.12-0.33) |
| Central Asia | 0.28 (0.13-0.43) | 0.24 (0.12-0.35) |
| North Africa and Middle East | 0.3 (0.14-0.45) | 0.26 (0.14-0.37) |
| South Asia | 0.11 (0.05-0.17) | 0.11 (0.05-0.17) |
| Southeast Asia | 0.12 (0.05-0.19) | 0.12 (0.05-0.18) |
| East Asia | 0.17 (0.07-0.26) | 0.15 (0.08-0.22) |
| Oceania | 0.2 (0.09-0.31) | 0.19 (0.09-0.29) |
| Western Sub-Saharan Africa | 0.18 (0.08-0.27) | 0.13 (0.07-0.19) |
| Eastern Sub-Saharan Africa | 0.12 (0.06-0.19) | 0.09 (0.05-0.13) |
| Central Sub-Saharan Africa | 0.16 (0.07-0.25) | 0.13 (0.07-0.19) |
| Southern Sub-Saharan Africa | 0.27 (0.12-0.42) | 0.25 (0.12-0.36) |

**Table S5.** Deaths of asthma in older adults attributable to high BMI in 1990 and 2021 for both sexes and all regions, with EAPC from 1990 to 2021.

| **Location** | **Death number**  **in 1990** | **Death number**  **in 2021** | **ASMR in 1990**  **(per 100,000)** | **ASMR in 2021**  **(per 100,000)** | **EAPC, 1990-2021**  **(%)** |
| --- | --- | --- | --- | --- | --- |
| **Global** |  |  |  |  |  |
| Global | 25799 (10789-41189) | 43628 (18366-71088) | 5.86 (2.45-9.36) | 4.14 (1.74-6.74) | -1.26 (-1.36--1.17) |
| Male | 11828  (5090-19781) | 17701  (7571-29364) | 6.4  (2.74-10.67) | 3.8  (1.63-6.3) | -1.86  (-1.95--1.76) |
| Female | 13972  (5876-23414) | 25927  (11044-43719) | 5.51  (2.32-9.25) | 4.41  (1.88-7.43) | -0.83  (-0.92--0.73) |
| **SDI regions** |  |  |  |  |  |
| High SDI | 6007 (2577-9474) | 3435 (1587-5455) | 4.17 (1.79-6.58) | 1.1 (0.51-1.74) | -5.03 (-5.47--4.58) |
| High-middle SDI | 5050 (2161-7929) | 4485 (2018-7137) | 4.53 (1.93-7.13) | 1.83 (0.82-2.91) | -3.5 (-3.77--3.23) |
| Middle SDI | 6232 (2561-10054) | 13429 (5908-21386) | 6.54 (2.69-10.59) | 4.51 (1.98-7.21) | -1.42 (-1.58--1.26) |
| Low-middle SDI | 6201 (2474-11037) | 17093 (6904-30212) | 10.28 (4.06-18.57) | 11.17 (4.51-19.96) | 0.46 (0.37-0.56) |
| Low SDI | 2269 (892-4113) | 5147 (1984-10039) | 10.3 (4.03-19.09) | 10.53 (4.05-20.83) | 0.18 (0.1-0.25) |
| **GBD regions** |  |  |  |  |  |
| Andean Latin America | 66 (28-112) | 78 (32-141) | 3 (1.26-5.1) | 1.12 (0.46-2.02) | -3.22 (-3.31--3.12) |
| Australasia | 150 (64-240) | 131 (59-208) | 4.99 (2.13-8.01) | 1.67 (0.75-2.63) | -3.73 (-4.56--2.89) |
| Caribbean | 125 (53-202) | 212 (92-359) | 4.1 (1.75-6.64) | 3.12 (1.36-5.3) | -1.26 (-1.52--1.01) |
| Central Asia | 865 (367-1395) | 672 (305-1058) | 16.43 (6.99-26.6) | 7.8 (3.53-12.29) | -3.09 (-3.62--2.56) |
| Central Europe | 1364 (590-2144) | 494 (225-773) | 7.79 (3.36-12.27) | 1.6 (0.73-2.51) | -5.72 (-6.25--5.19) |
| Central Latin America | 681 (299-1076) | 508 (233-796) | 8.11 (3.56-12.83) | 1.72 (0.79-2.7) | -5.49 (-5.69--5.28) |
| Central Sub-Saharan Africa | 247 (83-630) | 730 (238-2082) | 11.86 (3.8-32.81) | 15.54 (4.87-47.1) | 0.84 (0.78-0.9) |
| East Asia | 2476 (1019-4311) | 4046 (1818-6677) | 3.45 (1.41-6.15) | 1.72 (0.77-2.84) | -2.55 (-2.77--2.32) |
| Eastern Europe | 1877 (835-2940) | 384 (173-583) | 5.32 (2.36-8.33) | 0.81 (0.37-1.24) | -7.49 (-7.98--6.99) |
| Eastern Sub-Saharan Africa | 461 (183-830) | 1052 (385-2241) | 6.35 (2.49-11.97) | 6.52 (2.38-14.16) | -0.03 (-0.07-0.01) |
| High-income Asia Pacific | 1121 (479-1835) | 573 (245-990) | 5 (2.13-8.23) | 0.66 (0.28-1.13) | -7.84 (-8.34--7.34) |
| High-income North America | 885 (389-1396) | 734 (333-1122) | 1.88 (0.83-2.97) | 0.8 (0.37-1.23) | -3.5 (-3.83--3.17) |
| North Africa and Middle East | 3333 (1349-5524) | 5500 (2451-8731) | 20.65 (8.28-34.63) | 12.62 (5.59-20.12) | -1.78 (-1.89--1.66) |
| Oceania | 106 (43-194) | 234 (96-444) | 45.65 (18.87-84.1) | 36.91 (15.11-69.74) | -0.88 (-0.97--0.79) |
| South Asia | 4787 (1775-9305) | 17981 (7139-33959) | 8.53 (3.12-16.82) | 11.37 (4.49-21.75) | 1.24 (1.07-1.41) |
| Southeast Asia | 1993 (827-3392) | 4961 (2173-8152) | 7.9 (3.29-13.43) | 7.25 (3.19-11.91) | -0.42 (-0.57--0.27) |
| Southern Latin America | 181 (81-288) | 180 (81-280) | 3.2 (1.43-5.11) | 1.56 (0.7-2.42) | -2.63 (-2.98--2.28) |
| Southern Sub-Saharan Africa | 542 (219-1003) | 1274 (575-2038) | 19.64 (7.9-36.93) | 21.87 (9.8-35.14) | 0.17 (-0.4-0.74) |
| Tropical Latin America | 286 (128-465) | 498 (216-783) | 3.03 (1.35-4.96) | 1.61 (0.7-2.54) | -2.5 (-2.82--2.18) |
| Western Europe | 3290 (1425-5190) | 1588 (685-2510) | 4.24 (1.83-6.69) | 1.06 (0.46-1.66) | -4.98 (-5.54--4.43) |
| Western Sub-Saharan Africa | 963 (403-1599) | 1796 (777-3026) | 11.01 (4.62-18.25) | 9.94 (4.31-16.79) | -0.27 (-0.34--0.2) |
| **Abbreviations:** DALYs, disability-adjusted life years; ASMR, age-standardized mortality rates; EAPC, estimated annual percentage change | | | | | |

**Table S6.** DALYs of asthma in older adults attributable to high BMI in 1990 and 2021 for all nations, with EAPC from 1990 to 2021.

| **Location** | **DALY number**  **in 1990** | **DALY number**  **in 2021** | **ASDR in 1990**  **(per 100,000)** | **ASDR in 2021**  **(per 100,000)** | **EAPC, 1990-2021**  **(%)** |
| --- | --- | --- | --- | --- | --- |
| Afghanistan | 7152 (2766-13803) | 5499 (2069-11241) | 857.75 (329.38-1663.85) | 670.85 (252.73-1383.12) | -1.17 (-1.38--0.97) |
| Albania | 946 (409-1552) | 826 (357-1413) | 422.55 (182.4-695.1) | 143.93 (61.79-247.15) | -3.83 (-4.16--3.5) |
| Algeria | 4546 (1817-8056) | 10411 (4376-17705) | 369.96 (147.95-657.91) | 282.37 (117.33-487.44) | -0.91 (-0.95--0.86) |
| American Samoa | 13 (5-22) | 15 (7-26) | 601.99 (245.2-1048.86) | 307.62 (130-522.97) | -2.52 (-2.73--2.31) |
| Andorra | 10 (4-17) | 15 (6-25) | 136.66 (58.73-229.39) | 75.39 (31.95-126.55) | -2.1 (-2.33--1.88) |
| Angola | 849 (314-1927) | 2230 (799-4618) | 206.11 (73.87-493.12) | 185.27 (65.85-395.21) | -0.71 (-0.83--0.59) |
| Antigua and Barbuda | 7 (3-11) | 13 (6-20) | 96.07 (42.06-157.05) | 96.92 (43.81-155) | -0.52 (-0.83--0.21) |
| Argentina | 7663 (3395-12595) | 9629 (4335-15884) | 182.22 (80.58-299.79) | 133.65 (60.2-220.39) | -1.53 (-1.81--1.25) |
| Armenia | 211 (92-336) | 125 (57-193) | 63.32 (27.76-100.64) | 20.93 (9.53-32.48) | -3.43 (-3.93--2.93) |
| Australia | 5503 (2430-8905) | 6010 (2659-9699) | 213.7 (94.3-345.84) | 100.02 (44.19-161.5) | -2.54 (-2.75--2.34) |
| Austria | 3148 (1376-5119) | 1367 (588-2279) | 201.82 (88.24-328.19) | 60.51 (26.01-100.98) | -4.26 (-4.47--4.04) |
| Azerbaijan | 1811 (797-3008) | 1981 (882-3273) | 322.71 (140.55-547.47) | 167.85 (74.11-278.22) | -2.65 (-2.84--2.45) |
| Bahamas | 18 (8-29) | 38 (17-59) | 102.11 (44.9-163.38) | 76.48 (34.05-120.29) | -1.38 (-1.6--1.16) |
| Bahrain | 88 (37-147) | 196 (84-333) | 572.88 (237.98-973.69) | 285.67 (120.12-493.24) | -3.01 (-3.45--2.57) |
| Bangladesh | 7327 (2689-14658) | 17946 (6669-37152) | 135.76 (49.6-272.64) | 106.73 (39.73-221.18) | -0.82 (-0.97--0.67) |
| Barbados | 66 (28-106) | 104 (47-165) | 173.12 (75.55-279.16) | 149.43 (68.03-237.17) | -0.68 (-0.93--0.43) |
| Belarus | 3684 (1527-6221) | 1580 (650-2725) | 216.9 (89.89-366.77) | 71.28 (29.33-123.08) | -4.3 (-4.56--4.03) |
| Belgium | 3525 (1536-5786) | 1634 (707-2712) | 174.94 (76.31-287.16) | 55.27 (23.88-91.93) | -4.22 (-4.5--3.94) |
| Belize | 11 (5-18) | 32 (15-49) | 98.76 (43.67-160.89) | 91.25 (42.81-140.57) | -0.99 (-1.51--0.46) |
| Benin | 694 (273-1319) | 1197 (454-2488) | 297.1 (116.7-566.01) | 216.8 (81.35-454.93) | -0.93 (-1.17--0.7) |
| Bermuda | 9 (4-15) | 10 (5-15) | 121.44 (51.75-194.84) | 54.42 (24.87-84.44) | -3.12 (-3.4--2.83) |
| Bhutan | 93 (35-191) | 191 (73-400) | 348.22 (130.02-731.05) | 266.16 (101.97-560.25) | -1.04 (-1.12--0.96) |
| Bolivia (Plurinational State of) | 324 (126-588) | 578 (242-1020) | 89.44 (34.58-164.08) | 54.51 (22.64-96.59) | -1.68 (-1.73--1.64) |
| Bosnia and Herzegovina | 770 (321-1311) | 837 (363-1388) | 164.31 (67.85-280.88) | 98.77 (42.8-163.79) | -1.89 (-1.97--1.81) |
| Botswana | 286 (103-611) | 572 (228-1055) | 467.05 (165.95-1014.15) | 381.58 (150.54-719.01) | -0.56 (-0.77--0.36) |
| Brazil | 9512 (4209-15345) | 15998 (7090-25437) | 92.18 (40.71-149.04) | 51.23 (22.66-81.47) | -2.56 (-2.88--2.25) |
| Brunei Darussalam | 26 (11-45) | 66 (27-110) | 249.33 (103.8-437.33) | 184 (75.89-309.44) | -0.87 (-1.04--0.71) |
| Bulgaria | 2751 (1178-4490) | 833 (354-1415) | 169.2 (72.42-275.87) | 43.4 (18.47-73.78) | -5.21 (-5.5--4.92) |
| Burkina Faso | 480 (202-889) | 844 (331-1591) | 96.11 (39.88-180.95) | 83.03 (32.48-157.72) | -0.51 (-0.7--0.32) |
| Burundi | 384 (143-764) | 601 (212-1334) | 143.53 (53.3-288.55) | 115.69 (40.33-264.3) | -1.12 (-1.35--0.89) |
| Cabo Verde | 63 (23-122) | 69 (27-131) | 213.55 (79.08-410.54) | 132.07 (50.85-249.93) | -1.58 (-2.18--0.98) |
| Cambodia | 605 (232-1132) | 1577 (637-2934) | 122.65 (47.44-231.4) | 114 (45.87-215.3) | -0.35 (-0.4--0.3) |
| Cameroon | 2071 (859-3743) | 3952 (1604-7121) | 427.58 (176.3-776.15) | 307.7 (123.49-562.6) | -0.94 (-1.03--0.86) |
| Canada | 7227 (3093-12112) | 6631 (2867-11210) | 169.45 (72.5-284.05) | 67.97 (29.37-114.96) | -2.67 (-2.97--2.36) |
| Central African Republic | 326 (113-780) | 794 (260-2242) | 262.66 (87.87-678.55) | 358.35 (111.73-1076.39) | 0.93 (0.87-0.99) |
| Chad | 716 (270-1396) | 1274 (453-2533) | 220.76 (82.74-433.16) | 206.82 (73.3-414.74) | -0.16 (-0.24--0.08) |
| Chile | 3032 (1324-4995) | 6225 (2726-10376) | 248.91 (108.6-410.44) | 187.38 (82.05-312.33) | -1.14 (-1.31--0.98) |
| China | 58127 (24553-98840) | 94003 (41653-151878) | 67.1 (28.01-115.6) | 37.41 (16.58-60.55) | -2.26 (-2.44--2.08) |
| Colombia | 2485 (1107-4018) | 1734 (755-2864) | 124.39 (55.33-201.13) | 24.61 (10.71-40.62) | -6.13 (-6.43--5.82) |
| Comoros | 43 (15-83) | 108 (40-210) | 196.58 (69.7-394.41) | 199.13 (72.55-390.39) | -0.18 (-0.39-0.04) |
| Congo | 425 (143-1004) | 779 (306-1560) | 339.78 (111.85-832.66) | 285.1 (108.33-594.91) | -0.88 (-1.05--0.71) |
| Cook Islands | 5 (2-9) | 5 (2-9) | 381.05 (149.32-688.63) | 163.18 (67.57-292.3) | -2.95 (-3.11--2.8) |
| Costa Rica | 249 (107-417) | 443 (190-729) | 119.25 (51.42-199.67) | 62.84 (26.94-103.55) | -2.67 (-3.05--2.28) |
| Coted'Ivoire | 1142 (457-2106) | 2537 (984-4814) | 288.95 (114.79-539.03) | 227.46 (87.74-435.28) | -0.67 (-0.76--0.58) |
| Croatia | 2147 (957-3544) | 1024 (443-1688) | 288.96 (128.75-476.21) | 83.55 (36.14-137.95) | -4.57 (-4.87--4.28) |
| Cuba | 734 (303-1186) | 1686 (724-2711) | 57.95 (23.92-93.72) | 70.05 (30.12-112.51) | 0.51 (0.3-0.72) |
| Cyprus | 231 (91-396) | 397 (166-673) | 240.09 (92.76-417.54) | 151.25 (62.9-258.25) | -1.68 (-1.8--1.56) |
| Czechia | 1966 (875-3084) | 1265 (568-2017) | 106.68 (47.49-167.57) | 44.09 (19.75-70.46) | -2.92 (-3.31--2.54) |
| Democratic People's Republic of Korea | 1740 (677-3595) | 4791 (1787-9780) | 112.25 (42.54-242.51) | 128.09 (47.06-268.84) | 0.41 (0.38-0.44) |
| Democratic Republic of the Congo | 4041 (1330-10336) | 12693 (4030-36921) | 234.52 (74.94-632.92) | 344.64 (106.03-1048.86) | 1.29 (1.22-1.36) |
| Denmark | 1672 (719-2739) | 824 (360-1354) | 160.98 (69.19-264.11) | 54.32 (23.58-89.46) | -4.36 (-4.63--4.09) |
| Djibouti | 12 (5-23) | 51 (18-105) | 92.31 (37.42-176.04) | 80.08 (28.55-162.18) | -0.67 (-0.75--0.59) |
| Dominica | 17 (7-28) | 22 (10-37) | 216.04 (94.03-357.68) | 209.11 (93.24-346.09) | -0.34 (-0.44--0.23) |
| Dominican Republic | 600 (244-1027) | 1137 (477-2085) | 143.02 (58.17-245.61) | 94.04 (39.39-172.6) | -1.27 (-1.46--1.08) |
| Ecuador | 512 (214-848) | 432 (188-697) | 86.27 (35.98-143.41) | 21.96 (9.57-35.41) | -4.19 (-4.48--3.9) |
| Egypt | 18542 (7483-31606) | 21925 (10118-35312) | 685.98 (273.61-1194.98) | 346.14 (157.31-561.86) | -2.47 (-2.7--2.24) |
| El Salvador | 904 (368-1534) | 1115 (492-1899) | 256.8 (104.4-435.67) | 142.22 (62.95-241.65) | -2.48 (-2.69--2.27) |
| Equatorial Guinea | 78 (26-177) | 108 (40-220) | 357.93 (119.31-843.12) | 215.74 (79.39-445.48) | -2.19 (-2.42--1.96) |
| Eritrea | 119 (44-234) | 328 (121-660) | 106.9 (38.8-214.5) | 116.94 (42.76-238) | 0.25 (0.14-0.36) |
| Estonia | 556 (244-883) | 203 (87-331) | 207.9 (91.31-329.86) | 57.09 (24.48-93.29) | -4.66 (-4.87--4.44) |
| Eswatini | 238 (88-480) | 464 (184-851) | 820.68 (297.06-1681.74) | 790.45 (310.69-1459.56) | 0.14 (-0.28-0.57) |
| Ethiopia | 2738 (1071-4851) | 3242 (1288-5983) | 119.32 (46.76-213.5) | 68.12 (26.93-126.47) | -2.2 (-2.33--2.06) |
| Fiji | 625 (254-1078) | 1033 (451-1758) | 1914.4 (780.1-3329.54) | 1368.46 (592.12-2335.34) | -1.77 (-2.13--1.4) |
| Finland | 1347 (576-2262) | 1656 (707-2755) | 144.33 (61.63-242.59) | 97.98 (41.72-163.77) | -1.5 (-1.58--1.43) |
| France | 19030 (8211-31561) | 13773 (5881-23009) | 177.09 (76.42-293.84) | 76.31 (32.43-127.98) | -3.31 (-3.5--3.12) |
| Gabon | 259 (88-533) | 300 (116-589) | 379.23 (126.9-803.58) | 269.69 (103.64-542.1) | -1.26 (-1.39--1.14) |
| Gambia | 111 (44-216) | 289 (113-586) | 291.2 (112.94-569.72) | 272.27 (105.52-558.22) | -0.22 (-0.43-0) |
| Georgia | 2109 (881-3324) | 512 (222-817) | 265.17 (110.4-420.01) | 63.67 (27.69-101.51) | -4.23 (-4.97--3.48) |
| Germany | 48226 (21251-77396) | 13773 (6113-22975) | 295.9 (130.41-474.65) | 57.11 (25.34-95.59) | -5.49 (-5.84--5.14) |
| Ghana | 787 (310-1488) | 2789 (1075-5093) | 117.96 (46.48-224.65) | 161.36 (61.33-298.59) | 1.22 (1.11-1.33) |
| Greece | 2275 (987-3776) | 1667 (726-2822) | 113.85 (49.39-189.08) | 58.63 (25.46-99.56) | -2.38 (-2.52--2.23) |
| Greenland | 14 (6-24) | 14 (6-24) | 400.02 (173.83-670.2) | 161.02 (67.98-270.95) | -3.66 (-3.94--3.39) |
| Grenada | 10 (4-17) | 15 (6-24) | 113.88 (49.06-184.85) | 108.13 (46.09-173.2) | -0.48 (-0.73--0.24) |
| Guam | 16 (7-27) | 16 (7-28) | 236.16 (98.81-410.01) | 59.19 (25.89-100.46) | -4.23 (-4.38--4.08) |
| Guatemala | 967 (406-1570) | 889 (396-1404) | 269.6 (114.03-439.36) | 69.63 (31.08-109.95) | -4.9 (-5.16--4.64) |
| Guinea | 994 (375-1867) | 1443 (535-2936) | 247.68 (93.63-466.67) | 229.25 (84.33-469.89) | 0 (-0.18-0.17) |
| Guinea-Bissau | 163 (63-312) | 231 (90-444) | 363.59 (139.62-694.42) | 311.28 (118.39-610.4) | -0.3 (-0.49--0.12) |
| Guyana | 82 (34-133) | 86 (38-141) | 184.69 (77.37-300.1) | 108.54 (48.24-177.04) | -1.96 (-2.18--1.73) |
| Haiti | 515 (172-1270) | 1166 (372-2993) | 134.78 (44.38-341.22) | 142.26 (45.31-365.95) | 0.23 (0.17-0.28) |
| Honduras | 427 (167-807) | 1097 (448-1989) | 188.27 (72.82-363.45) | 151.19 (61.74-275.25) | -0.7 (-0.84--0.55) |
| Hungary | 2519 (1115-4014) | 1296 (567-2071) | 128.22 (56.78-204.77) | 49.48 (21.59-79.3) | -3.61 (-3.81--3.41) |
| Iceland | 76 (33-127) | 64 (27-107) | 206.68 (89.43-345.66) | 84.56 (35.99-140.81) | -3.33 (-3.48--3.18) |
| India | 84822 (32656-162073) | 324484 (128321-586657) | 171.21 (65.2-331.46) | 232.23 (91.58-423.48) | 1.33 (1.17-1.48) |
| Indonesia | 12743 (5332-22042) | 41687 (17613-71195) | 122.68 (51.74-212.25) | 157.37 (66.39-269.8) | 0.89 (0.7-1.08) |
| Iran (Islamic Republic of) | 8871 (3661-14396) | 17364 (7724-27104) | 314.65 (128.52-514.31) | 201.1 (89.02-314.75) | -1.81 (-2.02--1.6) |
| Iraq | 2723 (1102-4499) | 4842 (2053-7991) | 302.83 (122.52-501.01) | 196.09 (82.53-325.47) | -1.94 (-2.12--1.76) |
| Ireland | 1141 (510-1854) | 785 (344-1312) | 213.04 (95.22-346.14) | 77.62 (33.97-129.91) | -3.58 (-3.83--3.33) |
| Israel | 1352 (591-2186) | 1063 (467-1732) | 213.84 (93.53-345.53) | 65.62 (28.85-107.05) | -4.27 (-4.64--3.89) |
| Italy | 23682 (10138-39656) | 7959 (3428-13458) | 200.24 (85.64-335.18) | 43.43 (18.68-73.82) | -5.64 (-6.08--5.2) |
| Jamaica | 213 (95-350) | 352 (160-556) | 93.62 (41.56-153.38) | 90.12 (41.02-142.22) | -0.63 (-1.1--0.16) |
| Japan | 31510 (13558-52832) | 12647 (5395-21645) | 146.94 (63.27-246.11) | 24.41 (10.39-41.83) | -6.61 (-7.02--6.19) |
| Jordan | 408 (177-673) | 972 (427-1619) | 311.28 (133.79-519.66) | 132.67 (57.88-222.2) | -3.44 (-3.83--3.05) |
| Kazakhstan | 5457 (2320-9167) | 6926 (3134-11244) | 363.2 (154.37-611.71) | 325.97 (147.44-529.28) | -0.99 (-1.62--0.37) |
| Kenya | 1054 (339-3030) | 4382 (1420-13654) | 115.15 (36.61-337.54) | 177.25 (56.28-564.74) | 1.76 (1.57-1.95) |
| Kiribati | 33 (12-80) | 57 (22-112) | 884.52 (295.25-2245.33) | 837.83 (319.68-1701.01) | -0.28 (-0.38--0.18) |
| Kuwait | 146 (64-227) | 261 (114-412) | 276.52 (121.26-433.94) | 99.54 (44.13-156.6) | -3.58 (-4.29--2.86) |
| Kyrgyzstan | 839 (375-1356) | 433 (191-696) | 226.93 (100.45-367.8) | 76.48 (33.71-123.06) | -4.59 (-5.03--4.16) |
| Lao People's Democratic Republic | 559 (221-1051) | 928 (328-1864) | 240.64 (94.18-460.17) | 188.3 (66.1-384.31) | -0.97 (-1.06--0.88) |
| Latvia | 1201 (546-1877) | 313 (138-500) | 256.78 (116.92-401.61) | 60.27 (26.49-96.19) | -5.18 (-5.49--4.88) |
| Lebanon | 590 (241-1025) | 1218 (547-2025) | 236.97 (96.27-415.63) | 158.96 (71.44-264.25) | -1.28 (-1.35--1.21) |
| Lesotho | 425 (151-893) | 763 (279-1475) | 433.89 (152.59-935.47) | 611.27 (221.5-1201.07) | 1.7 (1.34-2.07) |
| Liberia | 478 (188-891) | 584 (229-1147) | 346.5 (136.28-646.1) | 275.29 (106.95-545.92) | -0.76 (-1--0.51) |
| Libya | 886 (367-1589) | 2171 (945-3743) | 425.08 (174.89-765.04) | 416.62 (180.05-721.19) | 0 (-0.16-0.16) |
| Lithuania | 863 (383-1369) | 330 (143-543) | 148.42 (65.8-235.45) | 44.27 (19.17-72.99) | -4.46 (-4.74--4.18) |
| Luxembourg | 158 (70-256) | 144 (62-240) | 222.81 (98.44-361.92) | 108.24 (46.42-180.41) | -2.66 (-2.82--2.5) |
| Madagascar | 1051 (387-2054) | 2797 (975-5882) | 188.27 (67.89-373.38) | 258.08 (88.24-549.94) | 0.84 (0.77-0.91) |
| Malawi | 549 (217-1036) | 1338 (499-2653) | 131.48 (51.44-252.36) | 168 (62.71-335.7) | 0.54 (0.35-0.74) |
| Malaysia | 2094 (855-3663) | 3270 (1318-5604) | 200.6 (81.78-352.05) | 97.93 (39.19-169.65) | -3.49 (-3.97--3) |
| Maldives | 16 (6-30) | 24 (10-42) | 163.54 (60.38-313.47) | 69.01 (28.65-124.26) | -3.31 (-3.51--3.1) |
| Mali | 1456 (557-2783) | 2694 (989-5697) | 319.05 (121.08-615.63) | 274.98 (100.79-581.94) | -0.42 (-0.6--0.24) |
| Malta | 105 (44-175) | 125 (53-210) | 190.04 (80.59-318.39) | 95.97 (40.47-161.83) | -2.65 (-2.8--2.5) |
| Marshall Islands | 14 (6-24) | 19 (7-34) | 818.46 (329.9-1465.77) | 597.72 (233.15-1062.85) | -1.03 (-1.12--0.95) |
| Mauritania | 514 (212-928) | 649 (263-1121) | 460.24 (187.03-837.19) | 267.68 (107.81-464.67) | -1.71 (-2--1.42) |
| Mauritius | 506 (217-824) | 419 (184-669) | 609.19 (261.81-997.48) | 180.67 (78.66-288.95) | -4.26 (-4.58--3.95) |
| Mexico | 9490 (4256-14810) | 6184 (2888-9550) | 207.81 (93.1-325.45) | 41.23 (19.15-63.74) | -5.66 (-5.86--5.47) |
| Micronesia (Federated States of) | 50 (19-95) | 44 (18-81) | 904.28 (335.2-1789.61) | 597.9 (237.78-1101.06) | -1.5 (-1.58--1.42) |
| Monaco | 7 (3-12) | 9 (4-15) | 79.88 (33.75-135.87) | 70.77 (29.96-119.84) | -0.4 (-0.44--0.35) |
| Mongolia | 393 (157-692) | 268 (116-434) | 322.66 (128.76-572.2) | 111.21 (48.14-180.11) | -4.35 (-4.67--4.03) |
| Montenegro | 40 (17-68) | 57 (25-95) | 52.1 (21.96-87.84) | 43.26 (19.05-71.73) | -0.71 (-0.81--0.61) |
| Morocco | 6174 (2402-11034) | 13316 (5411-23183) | 378.2 (146.38-681.9) | 338.08 (137.57-590.19) | -0.36 (-0.4--0.31) |
| Mozambique | 658 (262-1280) | 1720 (645-3462) | 99.66 (39.61-197.77) | 143.11 (52.79-291.8) | 1.44 (1.31-1.57) |
| Myanmar | 9592 (3746-18683) | 16067 (5961-30516) | 360.02 (141.17-702.73) | 285.05 (105.92-543.34) | -1.05 (-1.17--0.93) |
| Namibia | 343 (122-681) | 676 (259-1299) | 474.5 (166.13-980.65) | 466.76 (176.94-906.73) | -0.22 (-0.49-0.05) |
| Nauru | 4 (1-7) | 4 (1-8) | 786.65 (298.02-1491.89) | 721.79 (254.9-1530.66) | -0.37 (-0.81-0.08) |
| Nepal | 2638 (961-5545) | 7483 (2757-15042) | 242.14 (87.8-511.49) | 260.77 (96.04-525.89) | 0.47 (0.26-0.68) |
| Netherlands | 3536 (1521-5859) | 3666 (1594-6149) | 137.2 (59.05-227.59) | 80.09 (34.74-134.53) | -1.67 (-2.07--1.27) |
| New Zealand | 1453 (634-2365) | 1115 (498-1785) | 281.69 (122.89-458.52) | 100.1 (44.76-160.2) | -3.59 (-4.24--2.95) |
| Nicaragua | 280 (117-460) | 508 (220-847) | 164.49 (68.7-270.98) | 90.43 (39.04-151.27) | -2.43 (-2.72--2.13) |
| Niger | 684 (253-1309) | 1555 (554-3224) | 229.31 (84.46-446.02) | 172.9 (61.16-365.48) | -0.81 (-1.08--0.54) |
| Nigeria | 10820 (4587-17744) | 20684 (8640-34056) | 216.53 (91.34-356.56) | 218.46 (91.92-360.35) | -0.1 (-0.15--0.05) |
| Niue | 1 (1-3) | 1 (0-2) | 510.72 (209.33-934.09) | 360.54 (147.01-649.34) | -1.38 (-1.47--1.28) |
| North Macedonia | 1094 (467-1810) | 917 (390-1507) | 496.95 (211.48-822.89) | 217 (92.04-357.69) | -3.37 (-3.75--2.98) |
| Northern Mariana Islands | 5 (2-9) | 10 (4-18) | 410.72 (165.73-737.57) | 215.64 (88.45-373.37) | -2.34 (-2.58--2.1) |
| Norway | 3324 (1437-5488) | 972 (424-1599) | 372.24 (160.6-615.3) | 74.42 (32.44-122.64) | -5.95 (-6.39--5.5) |
| Oman | 195 (74-358) | 371 (158-631) | 281.6 (106.95-518.05) | 210.75 (86.44-366.16) | -0.56 (-0.75--0.37) |
| Pakistan | 13491 (5212-27909) | 31679 (11952-67956) | 207.76 (79.68-432.37) | 248.41 (93.4-541.91) | 0.47 (0.14-0.81) |
| Palau | 9 (4-16) | 14 (6-24) | 862.83 (362.11-1499.67) | 632.85 (270.72-1110.73) | -0.92 (-0.98--0.86) |
| Palestine | 359 (147-619) | 567 (256-914) | 368.59 (150.71-639.19) | 226.02 (101.6-366.26) | -1.77 (-2.08--1.46) |
| Panama | 220 (96-353) | 342 (159-540) | 125.2 (54.45-201.24) | 61.78 (28.77-97.41) | -2.81 (-3.19--2.41) |
| Papua New Guinea | 1249 (456-2553) | 3254 (1210-6876) | 667.39 (246.12-1366.6) | 661.43 (244.64-1407.54) | -0.01 (-0.06-0.05) |
| Paraguay | 208 (88-352) | 568 (248-932) | 80.02 (33.74-135.42) | 81.15 (35.42-133.53) | 0.1 (0.05-0.15) |
| Peru | 812 (339-1428) | 1399 (604-2452) | 59.06 (24.65-104.23) | 34.29 (14.81-60.13) | -2.07 (-2.22--1.92) |
| Philippines | 7177 (2971-11980) | 20574 (8570-34185) | 235.08 (96.39-395.23) | 221.6 (92.37-368.48) | -0.27 (-0.53-0) |
| Poland | 32202 (14227-52679) | 14908 (6533-25053) | 565.79 (249.87-924.24) | 151.46 (66.3-254.54) | -4.63 (-5.2--4.06) |
| Portugal | 4814 (2061-7906) | 4275 (1836-7243) | 259.36 (111.03-425.65) | 136.26 (58.55-231.19) | -2.05 (-2.33--1.78) |
| Puerto Rico | 949 (413-1520) | 933 (420-1465) | 206.34 (89.68-330.42) | 99.15 (44.52-155.27) | -3.37 (-3.76--2.98) |
| Qatar | 29 (12-50) | 92 (40-153) | 418.48 (163.84-723.23) | 163.76 (71.2-275.07) | -3.59 (-4.29--2.88) |
| Republic of Korea | 10699 (4420-18053) | 7429 (3139-13149) | 426.18 (173.41-729.07) | 63.42 (26.75-112.44) | -7.22 (-7.62--6.82) |
| Republic of Moldova | 1124 (503-1792) | 303 (137-477) | 195.8 (87.54-311.83) | 37.26 (16.87-58.57) | -6.26 (-6.65--5.87) |
| Romania | 7983 (3335-12876) | 3313 (1415-5540) | 225.98 (93.98-364.77) | 65.64 (27.99-109.8) | -4.14 (-4.31--3.97) |
| Russian Federation | 54189 (24591-84364) | 13741 (6251-21448) | 225.53 (102.07-350.78) | 41.57 (18.89-64.84) | -6.71 (-7.09--6.32) |
| Rwanda | 694 (282-1332) | 1149 (443-2475) | 213.21 (85.47-416.98) | 161.19 (61.45-354.41) | -1.68 (-1.97--1.4) |
| Saint Kitts and Nevis | 6 (3-10) | 7 (3-11) | 119.15 (49.42-194.36) | 79.11 (35.63-125.45) | -1.41 (-1.73--1.09) |
| Saint Lucia | 20 (9-33) | 30 (13-49) | 177.93 (77.64-292.67) | 100.99 (44.53-161.41) | -2.53 (-2.88--2.17) |
| Saint Vincent and the Grenadines | 11 (5-18) | 16 (7-25) | 119.21 (52.54-191.83) | 86.03 (38.41-138.82) | -1.33 (-1.81--0.84) |
| Samoa | 74 (28-152) | 86 (36-155) | 787.39 (293.51-1666.04) | 547.8 (228.68-990.99) | -1.16 (-1.22--1.11) |
| San Marino | 4 (2-7) | 7 (3-11) | 92.17 (39.61-155.34) | 75.46 (32.51-126.34) | -0.43 (-0.55--0.31) |
| Sao Tome and Principe | 49 (20-89) | 68 (27-128) | 627.1 (252.08-1142.11) | 576.86 (225.18-1095.94) | -0.34 (-0.44--0.25) |
| Saudi Arabia | 2694 (1082-4789) | 4679 (2118-7722) | 463.53 (185.33-827.1) | 310.12 (138.23-521.68) | -1.56 (-1.71--1.41) |
| Senegal | 948 (366-1739) | 1827 (698-3441) | 258.4 (99.42-475.24) | 211.7 (80.62-400.32) | -0.38 (-0.77-0) |
| Serbia | 3084 (1284-5254) | 2576 (1158-4209) | 237.41 (97.45-412.22) | 113.54 (51.02-185.42) | -3.16 (-3.46--2.85) |
| Seychelles | 16 (7-28) | 23 (9-40) | 224.74 (90.56-389.69) | 170.85 (69.31-302.45) | -0.96 (-1.13--0.79) |
| Sierra Leone | 550 (217-1039) | 832 (313-1702) | 231.71 (91.35-440.02) | 204.7 (76.36-422.66) | -0.14 (-0.32-0.04) |
| Singapore | 324 (145-542) | 392 (153-699) | 127.67 (56.94-213.73) | 35.08 (13.64-62.51) | -5.12 (-5.5--4.75) |
| Slovakia | 834 (361-1359) | 627 (267-1038) | 105.97 (45.74-173.04) | 48.58 (20.72-80.51) | -2.88 (-3.02--2.74) |
| Slovenia | 743 (316-1210) | 511 (215-872) | 236.34 (100.68-385.65) | 87.37 (36.66-149.4) | -3.7 (-3.91--3.5) |
| Solomon Islands | 60 (24-117) | 136 (53-261) | 433.87 (173.43-847.3) | 407.65 (159.61-788.93) | -0.15 (-0.21--0.09) |
| Somalia | 424 (157-809) | 1314 (457-2667) | 188.95 (69.59-366.94) | 199.53 (68.33-408.67) | 0.08 (0-0.16) |
| South Africa | 8191 (3333-15259) | 19105 (8572-29639) | 352.58 (142.86-663.63) | 364.25 (162.9-568.55) | -0.23 (-0.89-0.42) |
| South Sudan | 358 (138-699) | 443 (160-911) | 118.32 (45.21-234.25) | 113.54 (40.89-235.46) | -0.36 (-0.64--0.08) |
| Spain | 10460 (4722-17093) | 10524 (4591-17046) | 143.57 (64.71-234.69) | 80.11 (34.85-130.02) | -2.01 (-2.16--1.86) |
| Sri Lanka | 3273 (1287-5763) | 6579 (2532-12216) | 277.59 (108.54-490.8) | 194.25 (74.94-360.55) | -1.22 (-1.38--1.07) |
| Sudan | 6400 (2084-16307) | 8020 (3135-15470) | 610.96 (196.76-1616.43) | 402.49 (156.03-779.63) | -1.65 (-1.75--1.55) |
| Suriname | 27 (11-46) | 49 (20-85) | 87.84 (36.53-151.48) | 62.48 (25.46-108.45) | -1.39 (-1.59--1.19) |
| Sweden | 5740 (2455-9503) | 2543 (1071-4321) | 298.16 (127.28-495.29) | 92.32 (38.67-156.94) | -4.1 (-4.63--3.57) |
| Switzerland | 2447 (1026-3997) | 1449 (612-2449) | 184.34 (77.37-301.69) | 62.88 (26.5-106.51) | -3.77 (-4.05--3.48) |
| Syrian Arab Republic | 2504 (1024-4265) | 6120 (2759-10187) | 441.66 (180.67-756.47) | 427.43 (189.38-715.66) | -0.42 (-0.57--0.27) |
| Taiwan (Province of China) | 4975 (2116-8035) | 4824 (2006-8001) | 278.18 (118.49-451.02) | 85.38 (35.47-141.47) | -4.12 (-4.41--3.83) |
| Tajikistan | 1305 (530-2324) | 1372 (589-2320) | 421.24 (169.88-755.45) | 240.79 (102.68-410.3) | -2.03 (-2.32--1.73) |
| Thailand | 5533 (2231-9948) | 12779 (5371-22418) | 144.36 (57.69-260.61) | 91.5 (38.41-160.78) | -2.04 (-2.22--1.86) |
| Timor-Leste | 25 (10-49) | 103 (40-194) | 98.83 (38.58-198.86) | 99.06 (38.07-187.07) | 0.08 (-0.13-0.28) |
| Togo | 335 (133-634) | 1009 (381-2022) | 260.3 (102.45-493.94) | 263.32 (97.59-531.3) | 0.22 (0.08-0.36) |
| Tokelau | 1 (0-2) | 1 (0-1) | 543.44 (193.16-1056.35) | 358.55 (137.04-689.74) | -1.57 (-1.69--1.44) |
| Tonga | 33 (14-60) | 43 (19-73) | 528.59 (220.15-961.62) | 454.94 (204.37-776.71) | -0.42 (-0.56--0.27) |
| Trinidad and Tobago | 176 (75-279) | 231 (103-375) | 173.59 (73.81-275.56) | 89.39 (39.73-145.26) | -2.66 (-2.95--2.37) |
| Tunisia | 1740 (733-2987) | 3971 (1671-6999) | 309.13 (129.24-535.62) | 251.44 (105.2-448.46) | -0.95 (-1.05--0.85) |
| Turkey | 12438 (5191-21100) | 25338 (11204-41274) | 325.63 (133.57-555.88) | 225.95 (99.61-369.63) | -1.27 (-1.46--1.07) |
| Turkmenistan | 599 (250-1012) | 247 (105-424) | 264.89 (110.64-449.47) | 51.63 (21.75-88.37) | -6.16 (-6.64--5.69) |
| Tuvalu | 6 (2-10) | 6 (2-11) | 748.66 (291.16-1352.2) | 489.31 (188.57-905.51) | -1.42 (-1.48--1.36) |
| Uganda | 1007 (388-1990) | 2196 (758-5188) | 137.05 (52.56-273.83) | 139.49 (47.79-333.63) | -0.42 (-0.6--0.24) |
| Ukraine | 8478 (3665-13889) | 2034 (877-3385) | 87.92 (38-144.22) | 19.15 (8.26-31.88) | -5.91 (-6.29--5.53) |
| United Arab Emirates | 214 (89-366) | 1152 (510-1868) | 635.87 (260.57-1096.02) | 470.41 (196.17-779.03) | -0.02 (-0.5-0.46) |
| United Kingdom | 28465 (12745-46966) | 21570 (9467-35204) | 242.92 (108.72-401.39) | 128.89 (56.41-210.58) | -2.22 (-2.48--1.96) |
| United Republic of Tanzania | 1851 (702-3472) | 4884 (1796-10086) | 149.39 (55.9-288.21) | 177.68 (64.81-369.55) | 0.48 (0.43-0.53) |
| United States of America | 72223 (31420-120580) | 124747 (54774-209880) | 172.01 (74.7-287.39) | 158.45 (69.48-266.76) | 0.81 (0.14-1.48) |
| United States Virgin Islands | 16 (7-27) | 22 (9-37) | 168.92 (72.13-279.88) | 90.51 (37.85-150.91) | -2.14 (-2.37--1.91) |
| Uruguay | 1150 (499-1870) | 1037 (464-1658) | 221.75 (96.26-360.74) | 142.52 (63.95-228.09) | -1.73 (-1.84--1.62) |
| Uzbekistan | 8126 (3418-13445) | 6589 (2898-10680) | 600.05 (251.36-997.92) | 220.31 (96.76-357.29) | -4.33 (-5.02--3.63) |
| Vanuatu | 34 (12-75) | 87 (31-190) | 553.49 (193.56-1243.17) | 512.7 (182.94-1180.32) | -0.42 (-0.52--0.33) |
| Venezuela (Bolivarian Republic of) | 1197 (523-1926) | 2249 (1025-3550) | 110.25 (48.05-177.87) | 59.79 (27.3-94.48) | -2.72 (-3.09--2.35) |
| Viet Nam | 3513 (1422-6695) | 8805 (3346-16849) | 73.61 (29.46-140.68) | 77.9 (29.29-150.49) | 0.35 (0.24-0.47) |
| Yemen | 2162 (799-4435) | 5997 (2175-10927) | 408.98 (149.56-889.24) | 409.04 (147.31-749.87) | -0.26 (-0.45--0.06) |
| Zambia | 414 (162-782) | 1302 (450-2697) | 133.78 (52.21-255.36) | 181.58 (62.88-379.79) | 0.78 (0.62-0.94) |
| Zimbabwe | 1428 (541-2526) | 4672 (1747-8765) | 306.81 (116.26-547.76) | 611.42 (225.21-1150.55) | 2.81 (2.49-3.13) |
| Abbreviations: DALYs, disability-adjusted life years; ASDR, age-standardized DALYs (disability-adjusted life years) rate; EAPC, estimated annual percentage change | | | | | |

**Table S7.** Deaths of asthma in older adults attributable to high BMI in 1990 and 2021 for all nations, with EAPC from 1990 to 2021.

| **Location** | **Death number**  **in 1990** | **Death number**  **in 2021** | **ASMR in 1990**  **(per 100,000)** | **ASMR in 2021**  **(per 100,000)** | **EAPC, 1990-2021**  **(%)** |
| --- | --- | --- | --- | --- | --- |
| Afghanistan | 307 (115-609) | 257 (92-542) | 41.25 (15.27-82.24) | 32.18 (11.45-68.46) | -1.16 (-1.38--0.95) |
| Albania | 46 (20-76) | 39 (16-72) | 22.2 (9.47-37.23) | 7.23 (2.88-13.41) | -3.97 (-4.41--3.54) |
| Algeria | 213 (82-391) | 510 (208-919) | 21.73 (8.43-39.72) | 16.24 (6.46-29.62) | -0.76 (-0.82--0.69) |
| American Samoa | 1 (0-1) | 1 (0-1) | 34.71 (13.87-62.62) | 18.62 (7.63-32.48) | -2.35 (-2.57--2.13) |
| Andorra | 0 (0-0) | 0 (0-0) | 2.21 (0.81-4.28) | 0.75 (0.28-1.45) | -3.41 (-3.81--3) |
| Angola | 34 (12-83) | 92 (31-203) | 9.68 (3.17-25.62) | 9 (3.03-20.73) | -0.6 (-0.72--0.47) |
| Antigua and Barbuda | 0 (0-0) | 0 (0-1) | 3.54 (1.52-5.86) | 3.64 (1.62-5.95) | -0.59 (-1.03--0.14) |
| Argentina | 108 (48-175) | 94 (43-150) | 2.68 (1.18-4.37) | 1.28 (0.58-2.03) | -2.79 (-3.28--2.29) |
| Armenia | 6 (2-9) | 4 (2-6) | 1.86 (0.79-2.97) | 0.7 (0.31-1.09) | -3.26 (-3.6--2.93) |
| Australia | 125 (53-204) | 106 (48-171) | 5.02 (2.12-8.16) | 1.58 (0.71-2.54) | -3.85 (-4.67--3.03) |
| Austria | 74 (32-122) | 22 (9-36) | 4.62 (2-7.55) | 0.8 (0.35-1.31) | -6.35 (-6.9--5.8) |
| Azerbaijan | 72 (30-132) | 69 (29-121) | 13.97 (5.76-26.37) | 6.63 (2.8-11.65) | -2.8 (-2.99--2.6) |
| Bahamas | 1 (0-1) | 1 (1-2) | 3.42 (1.45-5.6) | 2.71 (1.15-4.48) | -1.41 (-1.74--1.07) |
| Bahrain | 4 (1-6) | 8 (3-14) | 29.61 (11.95-52.5) | 15.8 (6.37-28.47) | -2.73 (-3.22--2.25) |
| Bangladesh | 323 (114-663) | 797 (287-1709) | 6.3 (2.21-13.03) | 5.05 (1.82-10.84) | -0.81 (-1--0.61) |
| Barbados | 2 (1-3) | 3 (1-5) | 5.13 (2.16-8.29) | 4.22 (1.87-6.93) | -0.94 (-1.39--0.48) |
| Belarus | 75 (31-128) | 8 (4-14) | 4.69 (1.94-8) | 0.39 (0.17-0.64) | -9.6 (-10.43--8.77) |
| Belgium | 73 (31-119) | 31 (13-51) | 3.59 (1.53-5.88) | 0.85 (0.37-1.41) | -5.23 (-5.66--4.8) |
| Belize | 0 (0-1) | 1 (0-2) | 3.54 (1.52-5.96) | 3.24 (1.48-5.19) | -1.1 (-1.78--0.41) |
| Benin | 31 (12-61) | 53 (19-117) | 14.02 (5.36-27.68) | 10.72 (3.82-23.91) | -0.73 (-0.98--0.48) |
| Bermuda | 0 (0-1) | 0 (0-1) | 4.21 (1.76-6.96) | 1.71 (0.77-2.77) | -3.57 (-4.1--3.04) |
| Bhutan | 4 (1-8) | 9 (3-20) | 16.34 (5.62-36.57) | 13.52 (4.95-29.49) | -0.76 (-0.85--0.67) |
| Bolivia (Plurinational State of) | 13 (5-25) | 22 (8-43) | 3.99 (1.44-7.9) | 2.36 (0.88-4.54) | -1.73 (-1.78--1.67) |
| Bosnia and Herzegovina | 16 (6-31) | 14 (6-25) | 3.87 (1.44-7.43) | 1.74 (0.75-2.98) | -3.18 (-3.5--2.86) |
| Botswana | 13 (4-28) | 27 (10-51) | 24.44 (8.23-55.75) | 20.29 (7.67-40.73) | -0.42 (-0.66--0.18) |
| Brazil | 279 (125-455) | 480 (208-755) | 3.04 (1.36-4.98) | 1.59 (0.69-2.5) | -2.56 (-2.89--2.24) |
| Brunei Darussalam | 1 (0-2) | 2 (1-3) | 10.42 (4.01-19.69) | 5.34 (2.12-9.81) | -1.76 (-1.97--1.54) |
| Bulgaria | 58 (25-95) | 12 (5-19) | 4.48 (1.92-7.35) | 0.63 (0.27-1.02) | -7.38 (-7.92--6.84) |
| Burkina Faso | 20 (8-39) | 36 (13-70) | 4.59 (1.82-9.13) | 3.93 (1.48-7.79) | -0.53 (-0.71--0.35) |
| Burundi | 16 (6-34) | 24 (8-58) | 6.63 (2.35-14.13) | 5.39 (1.76-13.52) | -1.1 (-1.35--0.84) |
| Cabo Verde | 3 (1-7) | 4 (1-7) | 10.82 (3.9-21.85) | 6.89 (2.42-13.81) | -1.44 (-2.08--0.78) |
| Cambodia | 26 (10-50) | 70 (27-135) | 6.07 (2.34-11.79) | 5.83 (2.29-11.46) | -0.24 (-0.29--0.18) |
| Cameroon | 96 (39-176) | 184 (73-347) | 22.58 (9.17-41.87) | 16.62 (6.44-31.58) | -0.86 (-0.95--0.76) |
| Canada | 84 (37-136) | 62 (27-99) | 2.01 (0.87-3.26) | 0.59 (0.26-0.94) | -4.44 (-4.92--3.96) |
| Central African Republic | 13 (4-34) | 32 (10-97) | 12.6 (3.85-36.4) | 17.33 (4.89-57.33) | 0.97 (0.9-1.04) |
| Chad | 34 (12-67) | 57 (20-117) | 11.18 (4.05-22.41) | 10.51 (3.61-21.78) | -0.13 (-0.22--0.04) |
| Chile | 47 (21-75) | 60 (27-96) | 4.14 (1.83-6.6) | 1.8 (0.8-2.85) | -2.58 (-2.77--2.39) |
| China | 2224 (912-3918) | 3725 (1637-6172) | 3.2 (1.3-5.78) | 1.65 (0.73-2.73) | -2.45 (-2.69--2.2) |
| Colombia | 82 (37-133) | 40 (17-66) | 4.46 (1.98-7.23) | 0.55 (0.24-0.91) | -7.85 (-8.27--7.43) |
| Comoros | 2 (1-4) | 5 (2-10) | 9.48 (3.12-20.55) | 9.26 (3.18-19.63) | -0.31 (-0.55--0.06) |
| Congo | 17 (5-43) | 32 (11-70) | 15.7 (4.74-42.24) | 13.59 (4.59-31.3) | -0.77 (-0.91--0.62) |
| Cook Islands | 0 (0-0) | 0 (0-1) | 21.74 (8.07-41.04) | 9.85 (3.8-18.47) | -2.85 (-3.03--2.66) |
| Costa Rica | 7 (3-12) | 9 (4-15) | 3.56 (1.56-5.84) | 1.32 (0.57-2.12) | -3.95 (-4.66--3.25) |
| Coted'Ivoire | 47 (18-90) | 106 (40-211) | 14.66 (5.62-28.28) | 11.28 (4.21-22.51) | -0.7 (-0.79--0.62) |
| Croatia | 54 (24-88) | 21 (9-33) | 7.99 (3.5-13.09) | 1.58 (0.65-2.54) | -6.3 (-7.07--5.53) |
| Cuba | 23 (10-38) | 53 (23-88) | 1.89 (0.78-3.07) | 2.1 (0.89-3.47) | 0.15 (-0.25-0.54) |
| Cyprus | 7 (2-12) | 8 (3-14) | 9.27 (3.26-17.77) | 3.48 (1.39-6.53) | -3.57 (-3.86--3.29) |
| Czechia | 66 (29-105) | 34 (15-55) | 3.71 (1.64-5.88) | 1.14 (0.5-1.82) | -4.07 (-4.86--3.27) |
| Democratic People's Republic of Korea | 69 (24-170) | 198 (62-490) | 5.32 (1.74-13.75) | 5.79 (1.76-14.89) | 0.35 (0.3-0.41) |
| Democratic Republic of the Congo | 168 (53-456) | 557 (165-1765) | 11.6 (3.47-34.34) | 17.68 (4.99-59.24) | 1.41 (1.35-1.47) |
| Denmark | 39 (16-64) | 19 (8-31) | 3.56 (1.5-5.84) | 1.07 (0.46-1.78) | -5.22 (-5.84--4.59) |
| Djibouti | 0 (0-1) | 2 (1-4) | 4.32 (1.64-8.69) | 3.67 (1.24-7.85) | -0.71 (-0.81--0.62) |
| Dominica | 1 (0-1) | 1 (0-1) | 8.57 (3.46-14.93) | 8.21 (3.45-14.68) | -0.43 (-0.59--0.27) |
| Dominican Republic | 27 (11-48) | 48 (19-96) | 7.13 (2.82-12.6) | 4.05 (1.55-8.04) | -1.63 (-1.86--1.39) |
| Ecuador | 24 (10-41) | 12 (5-20) | 4.32 (1.78-7.37) | 0.64 (0.27-1.06) | -5.5 (-5.93--5.06) |
| Egypt | 815 (315-1453) | 827 (365-1412) | 36.3 (13.86-66.52) | 16.48 (7.14-28.21) | -2.65 (-2.87--2.42) |
| El Salvador | 35 (14-63) | 37 (15-68) | 10.16 (3.9-18.15) | 4.36 (1.74-8.08) | -3.45 (-3.74--3.17) |
| Equatorial Guinea | 3 (1-8) | 5 (2-10) | 16.74 (5.33-42.85) | 10.58 (3.61-23.09) | -2.05 (-2.29--1.81) |
| Eritrea | 4 (2-9) | 13 (4-27) | 4.68 (1.6-9.92) | 5.36 (1.84-11.46) | 0.46 (0.33-0.58) |
| Estonia | 19 (8-30) | 5 (2-7) | 7.14 (3.13-11.42) | 1.15 (0.49-1.87) | -6.41 (-6.72--6.1) |
| Eswatini | 11 (4-23) | 19 (7-37) | 41.86 (14.01-91.95) | 38.55 (13.72-74.43) | 0.11 (-0.31-0.54) |
| Ethiopia | 108 (42-196) | 139 (54-268) | 5.33 (2.05-9.81) | 3.16 (1.21-6.12) | -2.04 (-2.17--1.91) |
| Fiji | 31 (12-54) | 52 (22-90) | 111.59 (45.22-198.21) | 85.05 (36.16-147.02) | -1.62 (-2.03--1.21) |
| Finland | 25 (10-41) | 24 (10-39) | 2.67 (1.11-4.43) | 1.15 (0.49-1.89) | -3.15 (-3.38--2.92) |
| France | 340 (144-555) | 221 (94-365) | 3.02 (1.28-4.93) | 0.9 (0.39-1.49) | -4.69 (-5.09--4.29) |
| Gabon | 12 (4-26) | 13 (5-28) | 18.76 (5.76-43.83) | 13.66 (4.87-29.59) | -1.17 (-1.28--1.06) |
| Gambia | 5 (2-10) | 13 (5-28) | 13.97 (5.17-28.61) | 13.31 (4.79-28.99) | -0.11 (-0.33-0.12) |
| Georgia | 92 (38-147) | 22 (9-35) | 12.33 (5.07-19.9) | 2.65 (1.13-4.32) | -4.21 (-5.26--3.14) |
| Germany | 1336 (560-2146) | 262 (113-424) | 7.83 (3.28-12.58) | 0.9 (0.39-1.45) | -8.04 (-8.69--7.39) |
| Ghana | 33 (13-65) | 125 (46-237) | 5.86 (2.27-11.55) | 8.52 (3.05-16.47) | 1.45 (1.32-1.59) |
| Greece | 19 (8-31) | 10 (5-17) | 1 (0.44-1.62) | 0.27 (0.12-0.44) | -4.2 (-4.7--3.69) |
| Greenland | 0 (0-0) | 0 (0-0) | 7.47 (2.73-14.14) | 1.73 (0.7-3.12) | -5.41 (-5.71--5.11) |
| Grenada | 0 (0-1) | 0 (0-1) | 3.29 (1.39-5.46) | 3.38 (1.43-5.5) | -0.32 (-0.77-0.13) |
| Guam | 1 (0-1) | 1 (0-1) | 13.87 (5.65-25.32) | 2.74 (1.12-4.87) | -4.8 (-5.11--4.49) |
| Guatemala | 44 (19-73) | 37 (17-61) | 14.6 (6.18-23.94) | 3.2 (1.43-5.2) | -5.38 (-5.67--5.1) |
| Guinea | 44 (16-85) | 65 (23-140) | 12.02 (4.44-23.24) | 11.4 (3.98-24.67) | 0.14 (-0.06-0.33) |
| Guinea-Bissau | 7 (3-13) | 10 (4-19) | 17.35 (6.5-33.73) | 15.29 (5.52-31.39) | -0.18 (-0.39-0.02) |
| Guyana | 3 (1-5) | 3 (1-5) | 7.28 (2.97-12.12) | 4.02 (1.71-6.8) | -2.15 (-2.46--1.84) |
| Haiti | 19 (5-52) | 41 (11-122) | 5.6 (1.54-16.26) | 5.67 (1.56-16.74) | 0.09 (0.04-0.14) |
| Honduras | 19 (7-39) | 46 (17-89) | 9.27 (3.35-19.53) | 7.16 (2.72-13.69) | -0.83 (-1--0.66) |
| Hungary | 68 (29-109) | 26 (12-41) | 3.69 (1.54-5.92) | 0.95 (0.43-1.49) | -5.03 (-5.44--4.62) |
| Iceland | 1 (1-2) | 1 (0-1) | 3.16 (1.34-5.23) | 1.02 (0.45-1.69) | -4.23 (-4.67--3.8) |
| India | 3723 (1362-7397) | 15352 (6005-28831) | 8.51 (3.08-17.22) | 11.97 (4.65-22.77) | 1.5 (1.3-1.7) |
| Indonesia | 556 (234-978) | 1813 (769-3141) | 6.08 (2.58-10.7) | 7.99 (3.38-13.84) | 0.92 (0.72-1.11) |
| Iran (Islamic Republic of) | 335 (133-549) | 779 (332-1248) | 14.54 (5.74-24.01) | 9.65 (4.1-15.49) | -1.6 (-1.81--1.4) |
| Iraq | 108 (41-193) | 190 (73-334) | 12.34 (4.7-21.99) | 8.85 (3.4-15.65) | -1.71 (-1.92--1.5) |
| Ireland | 23 (10-37) | 11 (5-17) | 4.36 (1.91-7.04) | 0.98 (0.42-1.61) | -5.11 (-5.6--4.62) |
| Israel | 44 (19-72) | 30 (13-49) | 7.37 (3.18-12.01) | 1.72 (0.75-2.76) | -5.12 (-5.69--4.55) |
| Italy | 340 (147-540) | 106 (45-173) | 2.95 (1.27-4.7) | 0.43 (0.18-0.69) | -7.09 (-7.85--6.33) |
| Jamaica | 7 (3-12) | 12 (6-21) | 2.98 (1.27-5.1) | 3.09 (1.37-5.22) | -0.39 (-0.99-0.22) |
| Japan | 537 (237-869) | 234 (98-391) | 2.7 (1.19-4.37) | 0.31 (0.13-0.51) | -8.16 (-8.67--7.66) |
| Jordan | 16 (6-28) | 34 (14-62) | 14.01 (5.64-25.09) | 5.64 (2.32-10.19) | -3.76 (-4.23--3.29) |
| Kazakhstan | 251 (106-431) | 310 (141-513) | 17.82 (7.48-30.59) | 16.1 (7.31-26.62) | -0.97 (-1.63--0.32) |
| Kenya | 46 (14-146) | 191 (57-647) | 5.54 (1.63-18.08) | 8.87 (2.55-31.02) | 1.91 (1.72-2.11) |
| Kiribati | 1 (0-4) | 3 (1-5) | 46.11 (13.63-131.41) | 47.25 (16.97-102.57) | -0.02 (-0.12-0.09) |
| Kuwait | 6 (3-9) | 8 (4-13) | 13.03 (5.64-20.6) | 3.55 (1.62-5.62) | -4.52 (-5.46--3.57) |
| Kyrgyzstan | 28 (12-47) | 11 (5-18) | 8.29 (3.49-13.73) | 2.27 (0.97-3.64) | -5.34 (-5.87--4.81) |
| Lao People's Democratic Republic | 25 (10-48) | 44 (15-91) | 12.43 (4.71-24.49) | 10.01 (3.41-21.27) | -0.89 (-0.98--0.8) |
| Latvia | 39 (18-62) | 7 (3-12) | 8.5 (3.82-13.46) | 1.32 (0.58-2.1) | -6.6 (-6.99--6.21) |
| Lebanon | 19 (7-36) | 42 (18-73) | 8.48 (3.06-16.31) | 5.02 (2.12-8.77) | -1.54 (-1.67--1.4) |
| Lesotho | 21 (7-47) | 35 (13-70) | 23.41 (7.86-54.01) | 31.93 (11.24-64.42) | 1.63 (1.27-1.99) |
| Liberia | 21 (8-40) | 26 (10-53) | 16.88 (6.5-31.95) | 13.59 (5.04-28.11) | -0.68 (-0.96--0.4) |
| Libya | 41 (16-78) | 101 (42-185) | 20.52 (7.83-39.33) | 20.85 (8.54-38.16) | 0.25 (0.09-0.42) |
| Lithuania | 27 (12-43) | 7 (3-11) | 4.72 (2.08-7.51) | 0.84 (0.37-1.35) | -6.28 (-6.64--5.92) |
| Luxembourg | 4 (2-6) | 2 (1-3) | 5.34 (2.26-8.62) | 1.44 (0.61-2.32) | -4.71 (-5.16--4.26) |
| Madagascar | 47 (16-95) | 117 (39-260) | 9.49 (3.23-19.73) | 13.06 (4.2-29.48) | 0.87 (0.8-0.94) |
| Malawi | 23 (9-46) | 56 (20-119) | 6.54 (2.44-13.17) | 8 (2.84-16.96) | 0.43 (0.24-0.62) |
| Malaysia | 83 (32-148) | 134 (51-246) | 8.31 (3.22-14.99) | 4.57 (1.73-8.44) | -3.13 (-3.65--2.61) |
| Maldives | 1 (0-1) | 1 (0-2) | 7.79 (2.74-15.85) | 3.45 (1.39-6.48) | -3.11 (-3.34--2.88) |
| Mali | 63 (24-122) | 119 (43-258) | 15.97 (5.96-31.47) | 13.83 (4.99-29.74) | -0.39 (-0.58--0.2) |
| Malta | 2 (1-2) | 1 (1-2) | 2.94 (1.24-4.84) | 0.89 (0.38-1.5) | -4.63 (-5--4.25) |
| Marshall Islands | 1 (0-1) | 1 (0-2) | 45.55 (17.84-84.2) | 34.35 (12.99-62.33) | -0.96 (-1.05--0.87) |
| Mauritania | 22 (8-42) | 25 (9-47) | 21.71 (8.18-41.34) | 11.56 (4.27-21.57) | -1.96 (-2.28--1.64) |
| Mauritius | 24 (10-39) | 20 (8-32) | 32.09 (13.72-53.18) | 9.11 (3.92-14.92) | -4.32 (-4.66--3.98) |
| Mexico | 427 (192-677) | 236 (108-368) | 10.46 (4.71-16.63) | 1.67 (0.76-2.61) | -6.26 (-6.46--6.06) |
| Micronesia (Federated States of) | 2 (1-5) | 2 (1-4) | 49.05 (17.44-104.79) | 34.18 (13.19-64.93) | -1.34 (-1.42--1.26) |
| Monaco | 0 (0-0) | 0 (0-0) | 0.25 (0.1-0.48) | 0.22 (0.09-0.42) | -0.44 (-0.54--0.34) |
| Mongolia | 15 (6-29) | 8 (4-15) | 13.45 (4.97-25.69) | 4.02 (1.69-6.94) | -4.91 (-5.28--4.53) |
| Montenegro | 1 (0-1) | 1 (0-2) | 0.98 (0.39-1.8) | 0.78 (0.31-1.36) | -1.29 (-1.73--0.86) |
| Morocco | 305 (116-570) | 644 (258-1151) | 20.08 (7.59-38.02) | 17.94 (7.19-32.11) | -0.34 (-0.39--0.28) |
| Mozambique | 27 (10-55) | 69 (24-147) | 4.62 (1.76-9.77) | 6.57 (2.23-14.21) | 1.45 (1.32-1.59) |
| Myanmar | 434 (169-855) | 786 (290-1510) | 18.5 (7.23-36.5) | 15.38 (5.69-29.73) | -0.88 (-0.99--0.77) |
| Namibia | 15 (5-32) | 32 (12-64) | 25.31 (8.43-55.56) | 25.24 (9.18-50.86) | -0.13 (-0.4-0.14) |
| Nauru | 0 (0-0) | 0 (0-0) | 39.91 (14.51-77.3) | 42.09 (14.2-96.14) | 0.13 (-0.27-0.54) |
| Nepal | 113 (40-240) | 335 (121-693) | 11.34 (4-24.34) | 12.59 (4.57-26.13) | 0.58 (0.35-0.82) |
| Netherlands | 39 (16-63) | 34 (14-56) | 1.47 (0.63-2.41) | 0.65 (0.27-1.09) | -2.14 (-3.07--1.2) |
| New Zealand | 24 (11-39) | 25 (11-39) | 4.85 (2.1-7.81) | 2.13 (0.94-3.36) | -3.14 (-4.38--1.88) |
| Nicaragua | 10 (4-18) | 16 (6-29) | 6.36 (2.54-11.18) | 3.1 (1.2-5.63) | -2.76 (-3.12--2.4) |
| Niger | 28 (10-56) | 64 (22-141) | 11.41 (4.05-23.08) | 8.46 (2.85-18.95) | -0.84 (-1.14--0.54) |
| Nigeria | 424 (174-720) | 744 (304-1273) | 9.51 (3.89-16.23) | 8.98 (3.74-15.37) | -0.25 (-0.33--0.16) |
| Niue | 0 (0-0) | 0 (0-0) | 29.29 (11.28-55.98) | 21.98 (8.54-41.38) | -1.18 (-1.27--1.08) |
| North Macedonia | 27 (11-47) | 17 (7-31) | 13.29 (5.32-23.61) | 4.89 (1.92-9.3) | -4.24 (-4.61--3.87) |
| Northern Mariana Islands | 0 (0-0) | 1 (0-1) | 23.86 (9.3-44.01) | 13.59 (5.34-24.33) | -2.22 (-2.5--1.94) |
| Norway | 68 (30-109) | 18 (8-29) | 7.06 (3.09-11.32) | 1.21 (0.52-1.93) | -6.46 (-7.09--5.83) |
| Oman | 9 (3-16) | 15 (5-27) | 13.37 (4.82-25.48) | 9.92 (3.47-18.5) | -0.43 (-0.71--0.15) |
| Pakistan | 625 (229-1336) | 1487 (550-3362) | 10.34 (3.74-22.33) | 13.03 (4.77-30.15) | 0.65 (0.31-0.98) |
| Palau | 0 (0-1) | 1 (0-1) | 48.75 (19.94-86.68) | 39.3 (16.36-69.91) | -0.58 (-0.65--0.51) |
| Palestine | 15 (6-28) | 20 (9-35) | 17.61 (6.9-32.43) | 9.72 (4.24-16.73) | -2.1 (-2.53--1.67) |
| Panama | 9 (4-14) | 13 (6-21) | 5.21 (2.25-8.41) | 2.23 (1.01-3.6) | -3.28 (-3.81--2.75) |
| Papua New Guinea | 52 (18-109) | 145 (53-315) | 34.19 (12.25-71.6) | 34.89 (12.55-76.72) | 0.11 (0.05-0.17) |
| Paraguay | 7 (3-13) | 17 (7-31) | 2.83 (1.11-5.16) | 2.58 (1.03-4.67) | -0.22 (-0.34--0.1) |
| Peru | 29 (11-56) | 44 (16-86) | 2.2 (0.86-4.24) | 1.07 (0.39-2.1) | -2.69 (-2.92--2.45) |
| Philippines | 310 (125-531) | 888 (373-1490) | 11.86 (4.72-20.52) | 10.62 (4.48-17.86) | -0.35 (-0.62--0.08) |
| Poland | 620 (277-976) | 156 (71-245) | 11.73 (5.23-18.49) | 1.56 (0.71-2.44) | -7.4 (-8.25--6.55) |
| Portugal | 79 (35-128) | 39 (16-62) | 4.77 (2.09-7.72) | 0.97 (0.41-1.57) | -5.3 (-6.04--4.56) |
| Puerto Rico | 26 (11-41) | 25 (11-41) | 5.84 (2.55-9.39) | 2.36 (1.08-3.83) | -4.24 (-4.9--3.56) |
| Qatar | 1 (1-2) | 3 (1-5) | 22.74 (8.71-41.05) | 7.19 (2.83-13.08) | -4.5 (-5.37--3.63) |
| Republic of Korea | 574 (224-1005) | 333 (134-639) | 28.11 (10.87-49.76) | 3.01 (1.21-5.78) | -8.57 (-9.09--8.04) |
| Republic of Moldova | 31 (14-50) | 6 (3-9) | 5.99 (2.6-9.68) | 0.75 (0.33-1.18) | -7.73 (-8.23--7.24) |
| Romania | 232 (98-382) | 59 (25-95) | 7.3 (3.07-12.03) | 1.12 (0.48-1.8) | -6.29 (-6.65--5.93) |
| Russian Federation | 1533 (684-2395) | 327 (148-494) | 6.8 (3.02-10.63) | 1.03 (0.47-1.55) | -7.55 (-8.04--7.05) |
| Rwanda | 26 (10-53) | 38 (13-96) | 9.11 (3.43-19.33) | 6.3 (2.03-16.07) | -1.99 (-2.29--1.68) |
| Saint Kitts and Nevis | 0 (0-0) | 0 (0-0) | 4.57 (1.88-7.7) | 2.91 (1.3-4.76) | -1.54 (-2.01--1.06) |
| Saint Lucia | 1 (0-1) | 1 (0-2) | 6.99 (2.98-11.61) | 3.57 (1.54-5.98) | -3.19 (-3.69--2.68) |
| Saint Vincent and the Grenadines | 0 (0-1) | 1 (0-1) | 5.2 (2.31-8.56) | 3.42 (1.5-5.62) | -1.56 (-2.17--0.95) |
| Samoa | 4 (1-8) | 4 (2-8) | 43.06 (15.08-99.1) | 30.66 (12.19-57.76) | -1.1 (-1.16--1.04) |
| San Marino | 0 (0-0) | 0 (0-0) | 1.19 (0.48-2.2) | 0.55 (0.2-1.12) | -1.58 (-1.98--1.18) |
| Sao Tome and Principe | 2 (1-4) | 3 (1-6) | 32.58 (12.58-61.49) | 29.22 (10.25-58.02) | -0.35 (-0.41--0.29) |
| Saudi Arabia | 131 (50-238) | 194 (83-336) | 24.86 (9.5-45.42) | 16.27 (6.84-28.73) | -1.63 (-1.79--1.47) |
| Senegal | 43 (16-81) | 84 (31-163) | 13.08 (4.9-24.51) | 10.81 (3.98-21.13) | -0.34 (-0.75-0.07) |
| Serbia | 116 (45-220) | 90 (39-156) | 10.65 (4.04-20.48) | 3.98 (1.73-6.9) | -4.07 (-4.48--3.66) |
| Seychelles | 1 (0-1) | 1 (0-2) | 10.88 (4.3-19.62) | 8.93 (3.45-16.56) | -0.67 (-0.82--0.51) |
| Sierra Leone | 25 (10-49) | 37 (13-81) | 11.61 (4.46-22.59) | 10.13 (3.55-22.23) | -0.18 (-0.36-0) |
| Singapore | 9 (4-15) | 4 (2-7) | 3.99 (1.73-6.63) | 0.37 (0.15-0.62) | -8.75 (-9.23--8.27) |
| Slovakia | 26 (10-44) | 13 (5-22) | 3.41 (1.39-5.89) | 1.01 (0.42-1.79) | -4.51 (-4.83--4.19) |
| Slovenia | 13 (6-21) | 5 (2-8) | 4.32 (1.91-6.79) | 0.76 (0.34-1.23) | -6.49 (-7.12--5.85) |
| Solomon Islands | 2 (1-5) | 6 (2-12) | 22.25 (8.52-44.49) | 21.61 (8.26-43.57) | -0.03 (-0.08-0.02) |
| Somalia | 17 (6-34) | 49 (16-105) | 8.65 (2.98-17.75) | 8.77 (2.76-19) | -0.02 (-0.1-0.07) |
| South Africa | 418 (166-815) | 954 (427-1504) | 19.4 (7.69-38.25) | 20.11 (8.94-31.97) | -0.21 (-0.87-0.45) |
| South Sudan | 15 (6-32) | 18 (6-40) | 5.64 (2-11.75) | 5.32 (1.77-11.62) | -0.42 (-0.71--0.12) |
| Spain | 239 (105-384) | 294 (125-483) | 3.4 (1.49-5.46) | 1.7 (0.73-2.78) | -2.1 (-2.41--1.8) |
| Sri Lanka | 143 (54-257) | 255 (88-512) | 13.57 (5.11-24.53) | 8.29 (2.9-16.57) | -1.5 (-1.69--1.31) |
| Sudan | 304 (94-844) | 390 (144-782) | 32.2 (9.78-94.52) | 21.37 (7.81-43.08) | -1.62 (-1.71--1.52) |
| Suriname | 1 (0-2) | 2 (1-3) | 3.34 (1.31-6.01) | 2.25 (0.86-4.26) | -1.51 (-1.73--1.29) |
| Sweden | 80 (34-129) | 31 (13-52) | 3.78 (1.62-6.15) | 0.89 (0.38-1.48) | -4.52 (-5.09--3.94) |
| Switzerland | 58 (24-95) | 23 (10-38) | 4.08 (1.71-6.72) | 0.78 (0.34-1.3) | -5.77 (-6.34--5.2) |
| Syrian Arab Republic | 115 (46-203) | 262 (110-458) | 22.26 (9.01-39.47) | 21.69 (8.87-38.06) | -0.39 (-0.55--0.23) |
| Taiwan (Province of China) | 183 (79-296) | 123 (51-202) | 12.29 (5.29-20.02) | 2.15 (0.89-3.54) | -6.19 (-6.64--5.74) |
| Tajikistan | 61 (24-117) | 58 (24-103) | 20.74 (7.99-39.92) | 11.7 (4.77-20.88) | -2.08 (-2.44--1.71) |
| Thailand | 222 (83-420) | 549 (208-1035) | 6.42 (2.4-12.19) | 3.99 (1.51-7.53) | -2.11 (-2.31--1.91) |
| Timor-Leste | 1 (0-2) | 5 (2-9) | 5.12 (1.91-10.6) | 4.99 (1.87-9.68) | -0.03 (-0.23-0.18) |
| Togo | 15 (6-28) | 42 (15-88) | 12.98 (4.88-25.41) | 13.08 (4.48-27.58) | 0.22 (0.06-0.37) |
| Tokelau | 0 (0-0) | 0 (0-0) | 29.62 (9.98-59.93) | 21.15 (7.5-42.47) | -1.29 (-1.4--1.19) |
| Tonga | 1 (1-3) | 2 (1-4) | 25.14 (9.91-49.14) | 22.86 (9.61-41.35) | -0.19 (-0.36--0.02) |
| Trinidad and Tobago | 8 (3-13) | 10 (4-16) | 8.35 (3.55-13.52) | 3.97 (1.72-6.65) | -2.92 (-3.24--2.59) |
| Tunisia | 77 (32-138) | 185 (72-360) | 15.75 (6.4-28.58) | 12.75 (4.89-25.04) | -0.95 (-1.06--0.84) |
| Turkey | 414 (153-781) | 737 (305-1307) | 12.24 (4.47-23.29) | 7.16 (2.96-12.75) | -1.56 (-1.86--1.26) |
| Turkmenistan | 21 (9-37) | 9 (3-15) | 10.21 (4.16-17.97) | 1.96 (0.78-3.5) | -6.41 (-7.1--5.72) |
| Tuvalu | 0 (0-0) | 0 (0-1) | 39.82 (15.04-74.63) | 28.4 (10.55-54.87) | -1.13 (-1.19--1.07) |
| Uganda | 41 (15-86) | 87 (27-230) | 6.1 (2.17-13.1) | 6.11 (1.91-16.46) | -0.46 (-0.64--0.27) |
| Ukraine | 154 (65-256) | 25 (10-41) | 1.65 (0.7-2.76) | 0.23 (0.1-0.38) | -7.89 (-8.47--7.31) |
| United Arab Emirates | 7 (3-13) | 25 (10-44) | 23.9 (8.5-44.56) | 18.72 (6.55-33.47) | 1.11 (0.39-1.83) |
| United Kingdom | 399 (177-629) | 401 (175-628) | 3.29 (1.46-5.18) | 2.03 (0.89-3.17) | -1.5 (-2.07--0.93) |
| United Republic of Tanzania | 71 (25-142) | 186 (61-430) | 6.56 (2.27-13.87) | 7.49 (2.42-17.45) | 0.35 (0.31-0.39) |
| United States of America | 800 (353-1264) | 672 (306-1026) | 1.87 (0.83-2.95) | 0.83 (0.38-1.26) | -3.41 (-3.73--3.09) |
| United States Virgin Islands | 1 (0-1) | 1 (0-1) | 6.39 (2.52-11.26) | 2.64 (0.99-5.07) | -2.93 (-3.16--2.7) |
| Uruguay | 26 (11-42) | 25 (11-40) | 5.08 (2.18-8.22) | 3.06 (1.35-4.88) | -2.05 (-2.39--1.71) |
| Uzbekistan | 317 (123-553) | 182 (79-297) | 24.27 (9.41-42.5) | 6.98 (3.04-11.46) | -5.2 (-6.11--4.28) |
| Vanuatu | 2 (1-3) | 4 (1-9) | 30.3 (10.14-71.68) | 29.44 (10.15-73.57) | -0.23 (-0.34--0.12) |
| Venezuela (Bolivarian Republic of) | 47 (20-77) | 74 (33-124) | 4.7 (2-7.72) | 2.09 (0.94-3.49) | -3.54 (-4.03--3.05) |
| Viet Nam | 165 (62-325) | 389 (134-788) | 3.73 (1.39-7.41) | 3.82 (1.3-7.81) | 0.22 (0.14-0.31) |
| Yemen | 89 (31-200) | 263 (91-503) | 19.5 (6.7-48.05) | 20.3 (7-39.07) | -0.14 (-0.36-0.08) |
| Zambia | 18 (7-35) | 57 (19-122) | 6.47 (2.44-12.64) | 8.8 (2.95-19.17) | 0.86 (0.72-0.99) |
| Zimbabwe | 64 (24-117) | 206 (74-392) | 15.51 (5.81-28.47) | 30.33 (10.72-57.96) | 2.79 (2.49-3.1) |

**Table S8.** Decomposition analysis of the change in DALYs

| **location** | **Overall**  **Difference**  **(year)** | **Population**  **Structure**  **n (%)** | **Population**  **Growth**  **n (%)** | **Epidemiological**  **Change**  **n (%)** |
| --- | --- | --- | --- | --- |
| Global | 386552.03 | 14540.97 (3.76%) | 859568.61 (222.37%) | -487557.55 (-126.13%) |
| High SDI | -41236.12 | 1233.05 (-2.99%) | 195597.31 (-474.33%) | -238066.48 (577.33%) |
| High-middle SDI | -31645.54 | 3849.34 (-12.16%) | 138637.59 (-438.1%) | -174132.46 (550.26%) |
| Middle SDI | 164276.73 | 9227.2 (5.62%) | 232838.67 (141.74%) | -77789.14 (-47.35%) |
| Low-middle SDI | 232040.4 | 3897.52 (1.68%) | 213380.25 (91.96%) | 14762.63 (6.36%) |
| Low SDI | 63322.13 | 566.07 (0.89%) | 64597.9 (102.01%) | -1841.84 (-2.91%) |
| Andean Latin America | 761.19 | 32.98 (4.33%) | 2486.15 (326.61%) | -1757.94 (-230.95%) |
| Australasia | 168.59 | 119.94 (71.14%) | 6314.55 (3745.58%) | -6265.9 (-3716.72%) |
| Caribbean | 2548.92 | 16.74 (0.66%) | 3591.27 (140.89%) | -1059.09 -41.55%) |
| Central Asia | -2396.86 | -86.87 (3.62%) | 11670.41 (-486.9%) | -13980.4 (583.28%) |
| Central Europe | -28587.82 | 1140.09 (-3.99%) | 20534.16 (-71.83%) | -50262.07 (175.82%) |
| Central Latin America | -1659.26 | 496.84 (-29.94%) | 23138.43 (-1394.5%) | -25294.53 (1524.45%) |
| Central Sub-Saharan Africa | 10923.29 | 12.92 (0.12%) | 8797.88 (80.54%) | 2112.49 (19.34%) |
| East Asia | 38775.56 | 10035.8 (25.88%) | 87666.01 (226.09%) | -58926.24 (-151.97%) |
| Eastern Europe | -51590.22 | -430.86 (0.84%) | 13189.15 (-25.57%) | -64348.51 (124.73%) |
| Eastern Sub-Saharan Africa | 14513.72 | 16.49 (0.11%) | 13842.78 (95.38%) | 654.46 (4.51%) |
| High-income Asia Pacific | -22025.19 | 6494.87 (-29.49%) | 34145.76 (-155.03%) | -62665.81 (284.52%) |
| High-income North America | 51927.59 | 54.82 (0.11%) | 67363.31 (129.73%) | -15490.55 (-29.83%) |
| North Africa and Middle East | 55703.26 | 1868.56 (3.35%) | 109145.07 (195.94%) | -55310.37 (-99.29%) |
| Oceania | 2675.23 | 72.34 (2.7%) | 3261.26 (121.91%) | -658.36 (-24.61%) |
| South Asia | 273411.51 | 5112.79 (1.87%) | 216674.3 (79.25%) | 51624.42 (18.88%) |
| Southeast Asia | 67272.41 | 276.43 (0.41%) | 75579.4 (112.35%) | -8583.42 (-12.76%) |
| Southern Latin America | 5046.43 | 75.04 (1.49%) | 9294.81 (184.19%) | -4323.43 (-85.67%) |
| Southern Sub-Saharan Africa | 15338.73 | -254.39 (-1.66%) | 13413.8 (87.45%) | 2179.33 (14.21%) |
| Tropical Latin America | 6845.1 | 313.87 (4.59%) | 15218 (222.32%) | -8686.78 (-126.91%) |
| Western Europe | -74572.77 | 247.04 (-0.33%) | 61264.08 (-82.15%) | -136083.89 (182.48%) |
| Western Sub-Saharan Africa | 21472.63 | 108.36 (0.5%) | 24734.73 (115.19%) | -3370.46 (-15.7%) |

**Table S9.** Decomposition analysis of the change in death number

| **Location** | **Overall**  **Difference**  **(year)** | **Population**  **Structure**  **n (%)** | **Population**  **Growth**  **n (%)** | **Epidemiological**  **Change**  **n (%)** |
| --- | --- | --- | --- | --- |
| Global | 17828.36 | 2398.11 (13.45%) | 28220.55 (158.29%) | -12790.3 (-71.74%) |
| High SDI | -2572.11 | 572.16 (-22.24%) | 3524.41 (-137.02%) | -6668.68 (259.27%) |
| High-middle SDI | -564.81 | 576.62 (-102.09%) | 3794.55 (-671.83%) | -4935.97 (873.92%) |
| Middle SDI | 7196.8 | 1159.52 (16.11%) | 9834.26 (136.65%) | -3796.98 (-52.76%) |
| Low-middle SDI | 10891.02 | 553.38 (5.08%) | 9593.51 (88.09%) | 744.13 (6.83%) |
| Low SDI | 2878.79 | 129.49 (4.5%) | 2785.26 (96.75%) | -35.96 (-1.25%) |
| Andean Latin America | 12.2 | 4.42 (36.2%) | 94.33 (772.98%) | -86.54 (-709.18%) |
| Australasia | -18.45 | 20.94 (-113.5%) | 130.62 (-707.86%) | -170.02 (921.36%) |
| Caribbean | 86.85 | 9.87 (11.36%) | 124.38 (143.2%) | -47.39 (-54.57%) |
| Central Asia | -192.45 | -39.26 (20.4%) | 462.02 (-240.07%) | -615.2 (319.67%) |
| Central Europe | -870.06 | 152.06 (-17.48%) | 460.11 (-52.88%) | -1482.23 (170.36%) |
| Central Latin America | -172.46 | 67.36 (-39.06%) | 947.95 (-549.67%) | -1187.77 (688.73%) |
| Central Sub-Saharan Africa | 483.49 | 13.26 (2.74%) | 371.54 (76.85%) | 98.69 (20.41%) |
| East Asia | 1570.26 | 903.98 (57.57%) | 3426.05 (218.18%) | -2759.77 (-175.75%) |
| Eastern Europe | -1493.05 | 32.57 (-2.18%) | 342.34 (-22.93%) | -1867.96 (125.11%) |
| Eastern Sub-Saharan Africa | 591.26 | 17.3 (2.93%) | 562.18 (95.08%) | 11.77 (1.99%) |
| High-income Asia Pacific | -548.44 | 531.66 (-96.94%) | 982.2 (-179.09%) | -2062.31 (376.03%) |
| High-income North America | -150.97 | 8.6 (-5.7%) | 575.18 (-381%) | -734.75 (486.7%) |
| North Africa and Middle East | 2166.86 | 228.39 (10.54%) | 4561.67 (210.52%) | -2623.2 (-121.06%) |
| Oceania | 128.19 | 10.91 (8.51%) | 147.6 (115.14%) | -30.32 (-23.66%) |
| South Asia | 13193.69 | 677.79 (5.14%) | 9902.49 (75.05%) | 2613.42 (19.81%) |
| Southeast Asia | 2968.21 | 57.45 (1.94%) | 3305.14 (111.35%) | -394.38 (-13.29%) |
| Southern Latin America | -0.79 | 18.13 (-2301.67%) | 123.6 (-15688.24%) | -142.52 (18089.91%) |
| Southern Sub-Saharan Africa | 731.5 | -35.19 (-4.81%) | 658.41 (90.01%) | 108.29 (14.8%) |
| Tropical Latin America | 211.57 | 49.44 (23.37%) | 454.5 (214.82%) | -292.37 (-138.19%) |
| Western Europe | -1702.43 | 415.81 (-24.42%) | 1205.15 (-70.79%) | -3323.39 (195.21%) |
| Western Sub-Saharan Africa | 833.38 | 2.85 (0.34%) | 1016.06 (121.92%) | -185.53 (-22.26%) |

**Table S10.** Global asthma disability-adjusted life years attributable to high body mass index in older adults predicted to 2050 by the BAPC model across genders

| **Year** | **Predicted number**  **(Both)** | **Predicted number**  **(Male)** | **Predicted number**  **(Female)** |
| --- | --- | --- | --- |
| 2022 | 1259512 | 509096 | 750843 |
| 2023 | 1293392 | 524471 | 769829 |
| 2024 | 1330815 | 541439 | 790756 |
| 2025 | 1370652 | 559498 | 813005 |
| 2026 | 1411850 | 578201 | 835982 |
| 2027 | 1453294 | 597155 | 858975 |
| 2028 | 1495970 | 616881 | 882418 |
| 2029 | 1540893 | 637812 | 906905 |
| 2030 | 1587317 | 659525 | 932137 |
| 2031 | 1634582 | 681653 | 957849 |
| 2032 | 1681953 | 703857 | 983665 |
| 2033 | 1729917 | 726396 | 1009802 |
| 2034 | 1779310 | 749697 | 1036672 |
| 2035 | 1830085 | 773714 | 1064287 |
| 2036 | 1882240 | 798431 | 1092680 |
| 2037 | 1935748 | 823858 | 1121824 |
| 2038 | 1990861 | 850140 | 1151830 |
| 2039 | 2048039 | 877515 | 1182935 |
| 2040 | 2107736 | 906187 | 1215414 |
| 2041 | 2170489 | 936398 | 1249586 |
| 2042 | 2236889 | 968444 | 1285776 |
| 2043 | 2308141 | 1002897 | 1324637 |
| 2044 | 2384600 | 1039960 | 1366358 |
| 2045 | 2465721 | 1079398 | 1410662 |
| 2046 | 2551000 | 1120999 | 1457292 |
| 2047 | 2639930 | 1164557 | 1505973 |
| 2048 | 2734124 | 1210804 | 1557649 |
| 2049 | 2834881 | 1260388 | 1613035 |
| 2050 | 2941172 | 1312955 | 1671485 |

**Table S11.** Global number of asthma deaths attributable to high body mass index in older adults predicted to 2050 by the BAPC model across genders

| **Year** | **Predicted number**  **(Both)** | **Predicted number**  **(Male)** | **Predicted number**  **(Female)** |
| --- | --- | --- | --- |
| 2022 | 44995 | 18366 | 26695 |
| 2023 | 45948 | 18824 | 27219 |
| 2024 | 47025 | 19342 | 27810 |
| 2025 | 48173 | 19892 | 28440 |
| 2026 | 49356 | 20455 | 29093 |
| 2027 | 50539 | 21019 | 29744 |
| 2028 | 51776 | 21613 | 30417 |
| 2029 | 53112 | 22259 | 31140 |
| 2030 | 54512 | 22935 | 31898 |
| 2031 | 55954 | 23625 | 32684 |
| 2032 | 57409 | 24316 | 33481 |
| 2033 | 58917 | 25029 | 34309 |
| 2034 | 60517 | 25786 | 35188 |
| 2035 | 62190 | 26574 | 36110 |
| 2036 | 63928 | 27385 | 37073 |
| 2037 | 65716 | 28214 | 38068 |
| 2038 | 67589 | 29081 | 39111 |
| 2039 | 69580 | 30001 | 40221 |
| 2040 | 71677 | 30968 | 41392 |
| 2041 | 73880 | 31979 | 42627 |
| 2042 | 76189 | 33033 | 43925 |
| 2043 | 78656 | 34155 | 45314 |
| 2044 | 81315 | 35362 | 46814 |
| 2045 | 84149 | 36644 | 48418 |
| 2046 | 87147 | 37992 | 50123 |
| 2047 | 90303 | 39403 | 51926 |
| 2048 | 93682 | 40904 | 53866 |
| 2049 | 97341 | 42522 | 55973 |
| 2050 | 101252 | 44244 | 58237 |

**Table S12.** Global asthma age-standardized mortality rates attributable to high body mass index in older adults predicted to 2050 by the BAPC model across genders

| **Year** | **Predicted ASMR**  **(Both)** | **Predicted ASMR**  **(Male)** | **Predicted ASMR**  **(Female)** |
| --- | --- | --- | --- |
| 2022 | 4.10 | 3.78 | 4.36 |
| 2023 | 4.05 | 3.75 | 4.31 |
| 2024 | 4.01 | 3.72 | 4.26 |
| 2025 | 3.97 | 3.70 | 4.22 |
| 2026 | 3.93 | 3.67 | 4.17 |
| 2027 | 3.90 | 3.65 | 4.13 |
| 2028 | 3.86 | 3.63 | 4.09 |
| 2029 | 3.83 | 3.61 | 4.04 |
| 2030 | 3.80 | 3.59 | 4.01 |
| 2031 | 3.77 | 3.57 | 3.97 |
| 2032 | 3.74 | 3.56 | 3.94 |
| 2033 | 3.72 | 3.55 | 3.90 |
| 2034 | 3.69 | 3.53 | 3.87 |
| 2035 | 3.67 | 3.52 | 3.84 |
| 2036 | 3.65 | 3.51 | 3.81 |
| 2037 | 3.63 | 3.51 | 3.79 |
| 2038 | 3.62 | 3.50 | 3.76 |
| 2039 | 3.60 | 3.50 | 3.74 |
| 2040 | 3.59 | 3.49 | 3.72 |
| 2041 | 3.57 | 3.49 | 3.70 |
| 2042 | 3.56 | 3.49 | 3.68 |
| 2043 | 3.55 | 3.48 | 3.66 |
| 2044 | 3.54 | 3.48 | 3.64 |
| 2045 | 3.53 | 3.48 | 3.63 |
| 2046 | 3.52 | 3.48 | 3.61 |
| 2047 | 3.51 | 3.48 | 3.60 |
| 2048 | 3.50 | 3.48 | 3.58 |
| 2049 | 3.49 | 3.48 | 3.57 |
| 2050 | 3.49 | 3.49 | 3.55 |

**Table S13.** Global asthma age-standardized disability-adjusted life year rates attributable to high body mass index in older adults predicted to 2050 by the BAPC model across genders

| **Year** | **Predicted ASDR**  **(Both)** | **Predicted ASDR**  **(Male)** | **Predicted ASDR**  **(Female)** |
| --- | --- | --- | --- |
| 2022 | 112.21 | 99.17 | 123.52 |
| 2023 | 111.76 | 99.07 | 122.85 |
| 2024 | 111.37 | 99.02 | 122.23 |
| 2025 | 111.04 | 99.02 | 121.69 |
| 2026 | 110.79 | 99.07 | 121.23 |
| 2027 | 110.59 | 99.18 | 120.83 |
| 2028 | 110.45 | 99.33 | 120.48 |
| 2029 | 110.35 | 99.53 | 120.18 |
| 2030 | 110.30 | 99.77 | 119.94 |
| 2031 | 110.31 | 100.06 | 119.76 |
| 2032 | 110.36 | 100.38 | 119.62 |
| 2033 | 110.45 | 100.74 | 119.53 |
| 2034 | 110.57 | 101.13 | 119.46 |
| 2035 | 110.73 | 101.56 | 119.44 |
| 2036 | 110.93 | 102.01 | 119.45 |
| 2037 | 111.15 | 102.49 | 119.50 |
| 2038 | 111.41 | 103.00 | 119.57 |
| 2039 | 111.68 | 103.52 | 119.67 |
| 2040 | 111.98 | 104.07 | 119.79 |
| 2041 | 112.30 | 104.64 | 119.93 |
| 2042 | 112.63 | 105.23 | 120.09 |
| 2043 | 112.98 | 105.83 | 120.26 |
| 2044 | 113.35 | 106.44 | 120.45 |
| 2045 | 113.73 | 107.07 | 120.65 |
| 2046 | 114.12 | 107.71 | 120.86 |
| 2047 | 114.52 | 108.36 | 121.09 |
| 2048 | 114.93 | 109.03 | 121.32 |
| 2049 | 115.34 | 109.70 | 121.56 |
| 2050 | 115.77 | 110.37 | 121.80 |

**Table S14.** Global asthma mortality rate attributable to high body mass index in older adults in each age group predicted by the BAPC model to 2050

| **Year** | **Mortality rate**  **(60 to 64)** | **Mortality rate**  **(65 to 69)** | **Mortality rate**  **(70 to 74)** | **Mortality rate**  **(75 to 79)** | **Mortality rate**  **(80 to 84)** | **Mortality rate**  **(85 to 89)** | **Mortality rate**  **(90 to 94)** | **Mortality rate**  **(95 plus)** |
| --- | --- | --- | --- | --- | --- | --- | --- | --- |
| 2022 | 2.00 | 2.72 | 3.83 | 5.56 | 7.15 | 10.05 | 13.19 | 15.87 |
| 2023 | 1.99 | 2.72 | 3.79 | 5.43 | 7.07 | 9.90 | 13.12 | 15.77 |
| 2024 | 1.99 | 2.71 | 3.75 | 5.31 | 6.99 | 9.76 | 13.02 | 15.67 |
| 2025 | 1.99 | 2.70 | 3.72 | 5.20 | 6.90 | 9.62 | 12.89 | 15.57 |
| 2026 | 1.99 | 2.70 | 3.69 | 5.12 | 6.79 | 9.51 | 12.73 | 15.47 |
| 2027 | 1.98 | 2.70 | 3.68 | 5.06 | 6.66 | 9.41 | 12.56 | 15.40 |
| 2028 | 1.98 | 2.70 | 3.67 | 5.01 | 6.51 | 9.32 | 12.39 | 15.33 |
| 2029 | 1.98 | 2.69 | 3.67 | 4.97 | 6.37 | 9.23 | 12.22 | 15.24 |
| 2030 | 1.99 | 2.69 | 3.66 | 4.93 | 6.25 | 9.12 | 12.07 | 15.11 |
| 2031 | 1.99 | 2.70 | 3.67 | 4.91 | 6.16 | 8.99 | 11.94 | 14.94 |
| 2032 | 1.99 | 2.70 | 3.67 | 4.89 | 6.10 | 8.83 | 11.84 | 14.77 |
| 2033 | 2.00 | 2.70 | 3.67 | 4.89 | 6.06 | 8.65 | 11.75 | 14.59 |
| 2034 | 2.01 | 2.71 | 3.68 | 4.90 | 6.02 | 8.48 | 11.66 | 14.43 |
| 2035 | 2.02 | 2.72 | 3.69 | 4.91 | 5.99 | 8.34 | 11.55 | 14.28 |
| 2036 | 2.03 | 2.73 | 3.70 | 4.92 | 5.97 | 8.25 | 11.41 | 14.17 |
| 2037 | 2.04 | 2.74 | 3.71 | 4.94 | 5.97 | 8.19 | 11.24 | 14.09 |
| 2038 | 2.06 | 2.76 | 3.73 | 4.96 | 5.99 | 8.15 | 11.05 | 14.03 |
| 2039 | 2.08 | 2.78 | 3.75 | 4.98 | 6.01 | 8.12 | 10.86 | 13.96 |
| 2040 | 2.10 | 2.80 | 3.78 | 5.01 | 6.05 | 8.11 | 10.72 | 13.87 |
| 2041 | 2.12 | 2.83 | 3.81 | 5.04 | 6.08 | 8.12 | 10.64 | 13.76 |
| 2042 | 2.15 | 2.86 | 3.84 | 5.08 | 6.13 | 8.15 | 10.60 | 13.60 |
| 2043 | 2.18 | 2.89 | 3.88 | 5.13 | 6.18 | 8.20 | 10.59 | 13.42 |
| 2044 | 2.21 | 2.93 | 3.92 | 5.18 | 6.23 | 8.27 | 10.61 | 13.25 |
| 2045 | 2.25 | 2.97 | 3.97 | 5.23 | 6.30 | 8.35 | 10.64 | 13.14 |
| 2046 | 2.29 | 3.02 | 4.03 | 5.30 | 6.37 | 8.44 | 10.69 | 13.09 |
| 2047 | 2.34 | 3.07 | 4.09 | 5.37 | 6.45 | 8.55 | 10.79 | 13.11 |
| 2048 | 2.39 | 3.13 | 4.16 | 5.45 | 6.54 | 8.66 | 10.91 | 13.17 |
| 2049 | 2.45 | 3.20 | 4.23 | 5.54 | 6.64 | 8.78 | 11.06 | 13.25 |
| 2050 | 2.51 | 3.27 | 4.32 | 5.64 | 6.75 | 8.92 | 11.23 | 13.37 |

**Table S15.** Global asthma disability-adjusted life years rate attributable to high body mass index in older adults in each age group predicted by the BAPC model to 2050

| **Year** | **DALYs rate**  **(60 to 64)** | **DALYs rate**  **(65 to 69)** | **DALYs rate**  **(70 to 74)** | **DALYs rate**  **(75 to 79)** | **DALYs rate**  **(80 to 84)** | **DALYs rate**  **(85 to 89)** | **DALYs rate**  **(90 to 94)** | **DALYs rate**  **(95 plus)** |
| --- | --- | --- | --- | --- | --- | --- | --- | --- |
| 2022 | 93.84 | 104.62 | 117.61 | 129.89 | 125.59 | 139.64 | 162.78 | 192.28 |
| 2023 | 94.21 | 104.73 | 116.50 | 128.10 | 124.96 | 138.29 | 162.06 | 191.46 |
| 2024 | 94.59 | 105.03 | 115.53 | 126.25 | 124.30 | 137.02 | 161.17 | 190.67 |
| 2025 | 94.99 | 105.42 | 114.77 | 124.60 | 123.51 | 135.95 | 160.00 | 189.74 |
| 2026 | 95.42 | 105.86 | 114.29 | 123.21 | 122.48 | 135.08 | 158.62 | 188.81 |
| 2027 | 95.88 | 106.33 | 114.17 | 122.00 | 121.13 | 134.40 | 157.19 | 188.05 |
| 2028 | 96.36 | 106.82 | 114.37 | 120.93 | 119.55 | 133.81 | 155.78 | 187.35 |
| 2029 | 96.88 | 107.35 | 114.78 | 120.03 | 117.92 | 133.22 | 154.49 | 186.49 |
| 2030 | 97.45 | 107.91 | 115.33 | 119.35 | 116.49 | 132.50 | 153.43 | 185.32 |
| 2031 | 98.06 | 108.52 | 115.94 | 118.98 | 115.32 | 131.55 | 152.63 | 183.92 |
| 2032 | 98.72 | 109.17 | 116.60 | 119.01 | 114.33 | 130.26 | 152.04 | 182.50 |
| 2033 | 99.43 | 109.88 | 117.30 | 119.38 | 113.49 | 128.74 | 151.59 | 181.11 |
| 2034 | 100.21 | 110.64 | 118.06 | 120.00 | 112.82 | 127.18 | 151.15 | 179.89 |
| 2035 | 101.05 | 111.47 | 118.87 | 120.77 | 112.37 | 125.85 | 150.59 | 178.95 |
| 2036 | 101.97 | 112.37 | 119.76 | 121.63 | 112.23 | 124.81 | 149.78 | 178.34 |
| 2037 | 102.97 | 113.35 | 120.71 | 122.56 | 112.47 | 123.98 | 148.60 | 178.00 |
| 2038 | 104.05 | 114.41 | 121.75 | 123.56 | 113.06 | 123.33 | 147.18 | 177.85 |
| 2039 | 105.22 | 115.56 | 122.87 | 124.63 | 113.90 | 122.88 | 145.72 | 177.73 |
| 2040 | 106.49 | 116.81 | 124.08 | 125.79 | 114.91 | 122.68 | 144.54 | 177.49 |
| 2041 | 107.87 | 118.17 | 125.40 | 127.05 | 116.02 | 122.83 | 143.71 | 176.98 |
| 2042 | 109.37 | 119.64 | 126.83 | 128.40 | 117.21 | 123.43 | 143.14 | 176.06 |
| 2043 | 110.99 | 121.23 | 128.37 | 129.86 | 118.50 | 124.43 | 142.79 | 174.87 |
| 2044 | 112.75 | 122.96 | 130.05 | 131.45 | 119.88 | 125.72 | 142.68 | 173.65 |
| 2045 | 114.66 | 124.84 | 131.86 | 133.16 | 121.37 | 127.22 | 142.89 | 172.78 |
| 2046 | 116.72 | 126.87 | 133.83 | 135.01 | 122.98 | 128.87 | 143.54 | 172.35 |
| 2047 | 118.95 | 129.07 | 135.96 | 137.01 | 124.72 | 130.64 | 144.73 | 172.25 |
| 2048 | 121.37 | 131.45 | 138.26 | 139.18 | 126.59 | 132.55 | 146.42 | 172.44 |
| 2049 | 123.99 | 134.03 | 140.76 | 141.52 | 128.61 | 134.60 | 148.50 | 172.96 |
| 2050 | 126.83 | 136.82 | 143.46 | 144.05 | 130.79 | 136.80 | 150.86 | 173.89 |


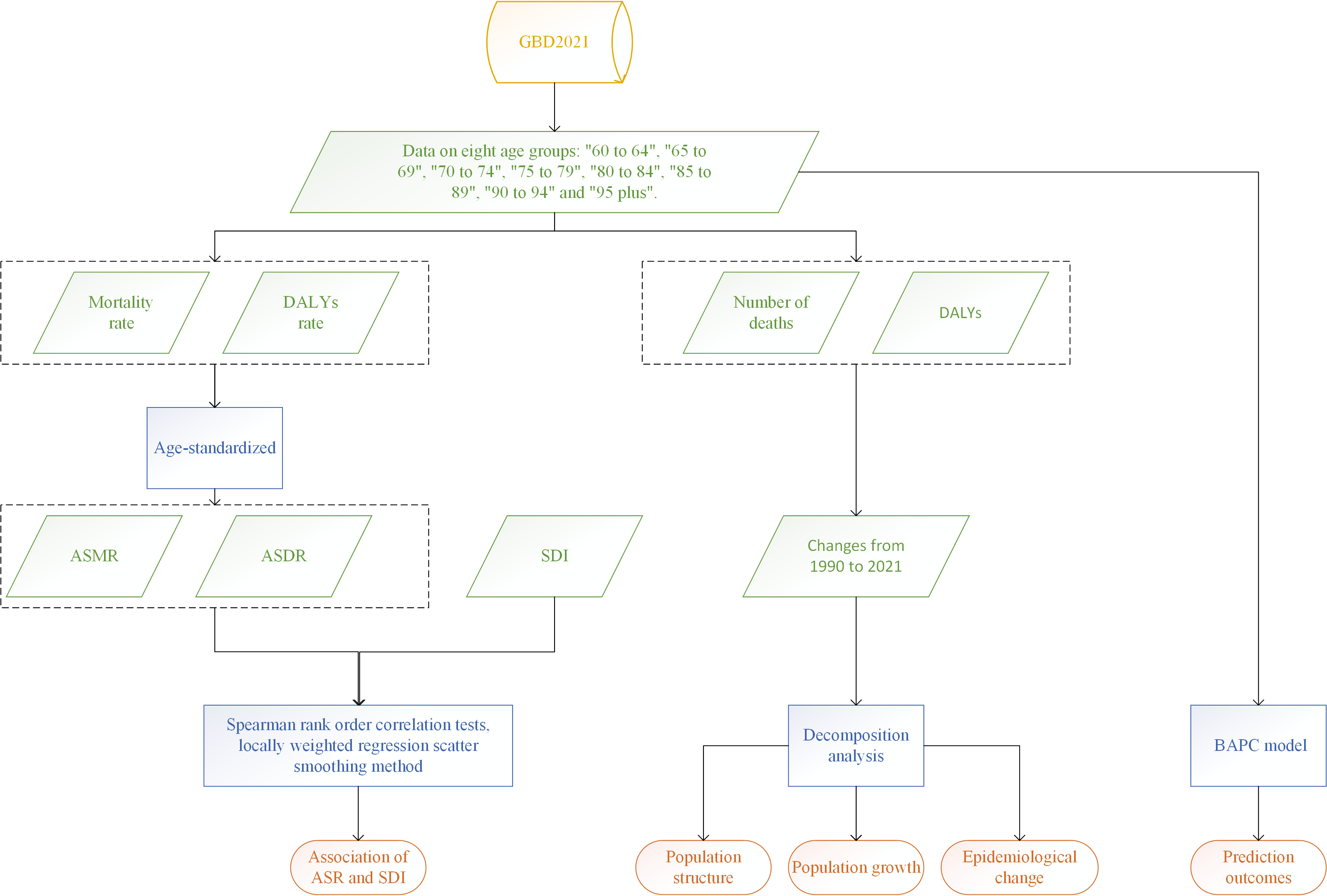


**Fig. S1.** Technical roadmap


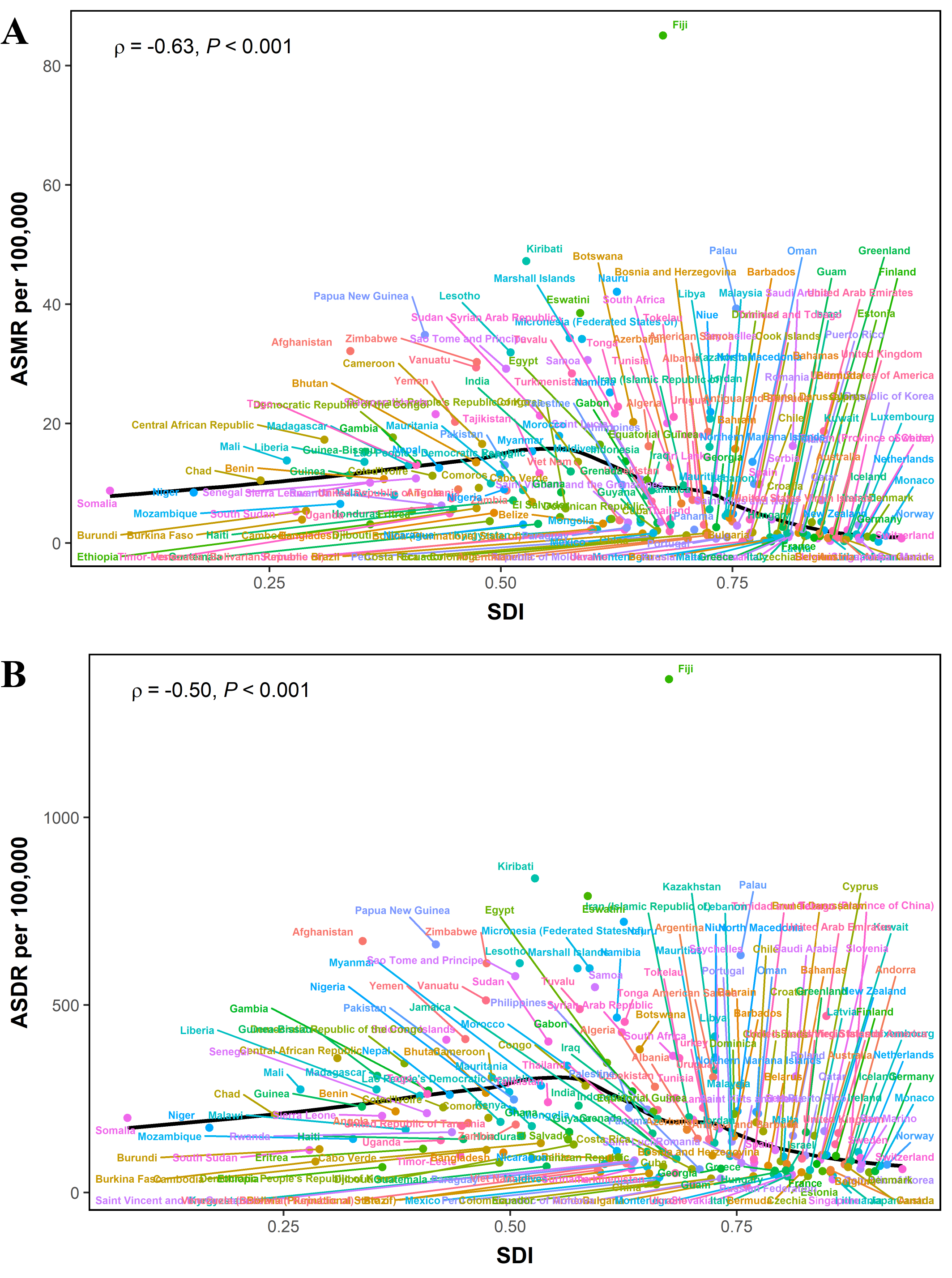


**Fig. S2.** Association of ASR of asthma in older adults attributable to high BMI with SDI at national level. ASMR (A) and ASDR (B) of asthma in older adults attributable to high BMI at national level, by SDI, in 2021. The black line in the figure represents the expectation ASR based on SDI. Abbreviations: ASMR, age-standardized mortality rate; ASDR, age-standardized DALYs (disability-adjusted life years) rate; ASR, age-standardized rate; SDI, Socio-demographic Index.
